# Clinical, pathological, and PAM50 gene expression features of HER2-low breast cancer

**DOI:** 10.1038/s41523-020-00208-2

**Published:** 2021-01-04

**Authors:** Francesco Schettini, Nuria Chic, Fara Brasó-Maristany, Laia Paré, Tomás Pascual, Benedetta Conte, Olga Martínez-Sáez, Barbara Adamo, Maria Vidal, Esther Barnadas, Aranzazu Fernández-Martinez, Blanca González-Farre, Esther Sanfeliu, Juan Miguel Cejalvo, Giuseppe Perrone, Giovanna Sabarese, Francesca Zalfa, Vicente Peg, Roberta Fasani, Patricia Villagrasa, Joaquín Gavilá, Carlos H. Barrios, Ana Lluch, Miguel Martín, Mariavittoria Locci, Sabino De Placido, Aleix Prat

**Affiliations:** 1 Department of Clinical Medicine and Surgery, University of Naples Federico II, Naples, Italy; 2 Translational Genomics and Targeted Therapeutics in Solid Tumors, August Pi i Sunyer Biomedical Research Institute, Barcelona, Spain; 3 SOLTI Breast Cancer Research Group, Barcelona, Spain; 4 Department of Medical Oncology, Hospital Clínic, Barcelona, Spain; 5 Department of Genetics, University of North Carolina, Chapel Hill, NC USA; 6 Department of Medical Oncology, Ospedale Policlinico San Martino, University of Genova, Genova, Italy; 7 Department of Pathology, Hospital Clínic, Barcelona, Spain; 8 INCLIVA Biomedical Research Institute, Hospital Clínico Universitario Valencia, University of Valencia, Valencia, Spain; 9 Pathology Department, Campus Bio-Medico University, Rome, Italy; 10 Vall d´Hebron University Hospital, Barcelona, Spain; 11 GEICAM, Grupo Español de Investigación en Cáncer de Mama, Madrid, Spain; 12 Vall d’Hebron Institute of Oncology (VHIO), Barcelona, Spain; 13 Instituto Valenciano de Oncología (IVO), Valencia, Spain; 14 Centro de Pesquisa Clínica Hospital São Lucas da PUCRS, Porto Alegre, Brazil; 15 LACOG, Latin American Cooperative Oncology Group, Porto Alegre, Brazil; 16 Hospital Universitario Clínico Valencia, Valencia, Spain; 17 Biomedical Research Centre Network in Cancer (CIBERONC), Valencia, Spain; 18 Hospital Gregorio Marañon, Madrid, Spain; 19 Department of Neuroscience, Reproductive Sciences and Dentistry, University of Naples Federico II, Naples, Italy

**Keywords:** Breast cancer, Breast cancer, Translational research, Cancer genomics, Breast cancer

## Abstract

Novel antibody-drug conjugates against HER2 are showing high activity in HER2-negative breast cancer (BC) with low HER2 expression (i.e., 1+ or 2+ and lack of *ERBB2* amplification). However, the clinical and molecular features of HER2-low BC are yet to be elucidated. Here, we collected retrospective clinicopathological and PAM50 data from 3,689 patients with HER2-negative disease and made the following observations. First, the proportion of HER2-low was higher in HR-positive disease (65.4%) than triple-negative BC (TNBC, 36.6%). Second, within HR-positive disease, *ERBB2* and luminal-related genes were more expressed in HER2-low than HER2 0. In contrast, no gene was found differentially expressed in TNBC according to HER2 expression. Third, within HER2-low, *ERBB2* levels were higher in HR-positive disease than TNBC. Fourth, HER2-low was not associated with overall survival in HR-positive disease and TNBC. Finally, the reproducibility of HER2-low among pathologists was suboptimal. This study emphasizes the large biological heterogeneity of HER2-low BC, and the need to implement reproducible and sensitive assays to measure low HER2 expression.

## Introduction

HER2-positive breast cancer is currently defined according to the ASCO/CAP guidelines using immunohistochemistry (IHC) and/or in situ hybridization (ISH)-based techniques^1,2^. These guidelines identify a tumor as HER2-positive when there is a complete and intense circumferential HER2 IHC staining in ≥10% of cells (score 3+) and/or the gene is amplified with an HER2/CEP17 ratio ≥2.0 and an average HER2 gene (*ERBB2*) copy number ≥4.0 signals/cell using ISH-based techniques^1^. In breast cancer, 10–20% of tumors are HER2-positive and 80–90% are HER2-negative^3,4^.

Within HER2-negative disease, substantial heterogeneity exists regarding the expression of hormone receptors (HR) and HER2. For example, HER2-negative tumors can express some protein level of HER2 by IHC^5^ (i.e., 1+ or 2+ and lack of *ERBB2* amplification by in situ hybridization techniques) and are identified as HER2-low. Traditionally, patients with HER2-low-expressing tumors do not seem to benefit from HER2-targeted therapies, such as 1-year of adjuvant trastuzumab^6^. However, two HER2-directed antibody-drug conjugates (ADC) with chemotherapeutics, namely trastuzumab deruxtecan (T-DXd) and trastuzumab duocarmazine (SYD985) have shown very promising therapeutic activity in patients with HER2-low breast cancer^7–9^. A large pivotal randomized phase III trial of T-DXd in patients with pretreated HER2-low metastatic breast cancer is underway (i.e., NCT03734029/DESTINY-Breast04).

Owing to the recent and increased interest in the HER2-low group, there is an urgent need to better understand its clinicopathological and molecular features. Thus, we decided to collect clinicopathological and PAM50 gene expression data from multiple datasets^10–17^ of HER2-negative disease and compare many features between HER2-low and HER2 0. Analyses were focused on the overall population and according to hormone receptor (HR) status and HER2 IHC expression.

## Results

### Clinicopathological characteristics of HER2-low disease

**Fig. 1:**
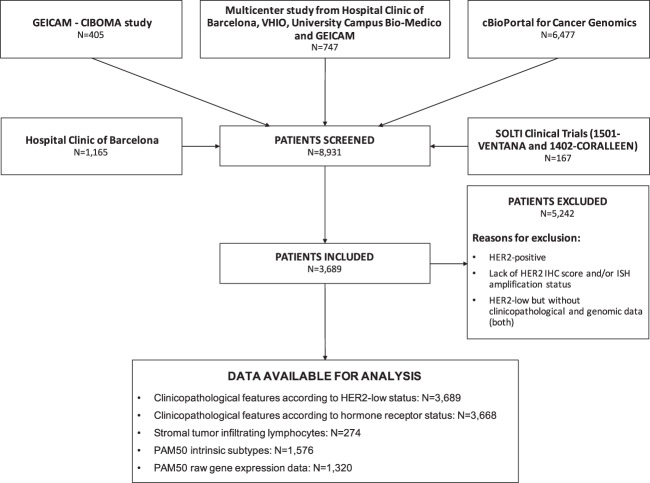
STROBE flow-chart. Flow-chart resuming the patient selection process, showing causes for exclusion and the number of patients with available data for the main analyses presented in the study. GEICAM Grupo Español de Investigación en Cáncer de Mama, CIBOMA Coalición Iberoamericana de Investigación en Oncología Mamaria, VHIO Vall d’Hebron Institute of Oncology, SOLTI Solid Tumor Intensification Group, IHC immunohistochemistry, ISH in-situ hybridization, HR hormone receptors.

Thirteen independent datasets for a total of 3,689 patients with HER2-negative breast cancer were explored (Fig. 1). Overall, 1,486 (40.3%) patients had HER2 0 tumors, 1,489 (40.4%) had HER2 1+ tumors and 714 (19.3%) had HER2 2+ tumors. Clinicopathological and gene expression data (when available) were largely obtained from primary disease (71.1% in HER2-low and 73.7% in HER2 0). According to HR status, 2,962 (80.8%) patients had HR-positive disease and 706 (19.2%) had triple-negative breast cancer (TNBC).

**Fig. 2:**
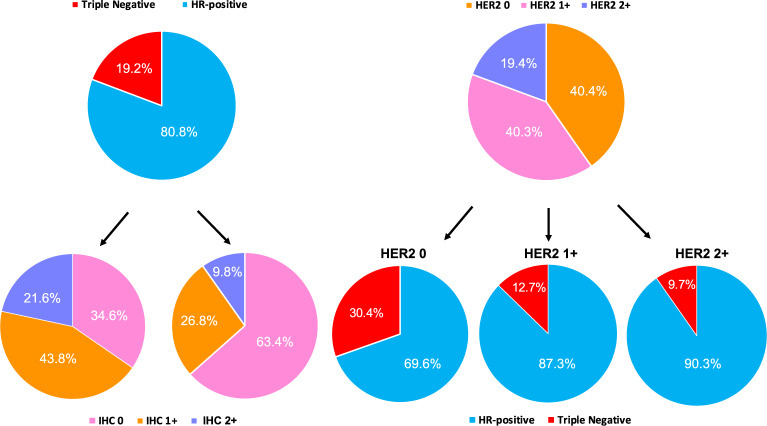
Hormone receptor status, HER2-low status, and IHC scores distributions within the HER2-negative population. HR hormone receptors, IHC immunohistochemistry, ISH in situ hybridization (including either FISH, SISH, and CISH).

HER2-low tumors were more frequently found within HR-positive disease compared to TNBC (65.4% vs. 36.5%, *p* < 0.001; Fig. 2). More specifically, HR-positive disease was characterized by higher rates of IHC 1+ and 2+ tumors, compared to TNBC (43.8% vs. 26.8% and 21.6% vs. 9.8%, respectively, *p* < 0.001; Fig. 2). In terms of other clinicopathological variables, HER2-low tumors presented larger primary tumor sizes (*p* = 0.007) and more nodal involvement (*p* = 0.010) compared to HER2 0 tumors (Table 1 and Supplementary Table 1). No male patient was observed within the HER2 0 cohort, compared to the 15 cases observed in the HER2-low subset (*p* = 0.001). The median age at diagnosis was higher for the HER2-low tumors compared to HER2 0 (59 vs. 55 years, *p* = 0.003). No statistically significant differences were observed in terms of menopausal status (p = 0.898), histological grade (*p* = 0.175), Ki67 IHC scores (*p* = 0.092 using a 14% cut-off) and percentage of stromal tumor-infiltrating lymphocytes (TILs) (*p* = 0.218), although TILs’ levels were differently distributed according to HER2 IHC levels (*p* = 0.033) and were higher in HER2 2+ (median: 5; interquartile range [IQR] 1–5) compared to 1+ (median: 1; IQR 1–5; *p* = 0.035) and 0 (median: 1; IQR 1–5; *p* = 0.035).

**Table 1:** Population characteristics according to HER2 status.

Demographics	HER2-negative						*p*^a^
	HER2 0		HER2-low		Overall population		
	*N*	%	*N*	%	*N*	%	
	1,486	40.3	2,203	59.7	3,689	100	
Age at diagnosis (years)							
Median	55		59		58		0.003
IQR	46–65		49–67		48–67		
Min–max	24–93		26–96		24–96		
Pts with available data	259	27.4	685	72.6	944	100	
Sex							
Male	0	0	15	0.7	15	0.4	0.001
Female	1,486	100	2,187	99.3	3,673	99.6	
Total	1,486	40.3	2,202	59.7	3,688	100	
Menopaual status							
Pre/perimenopausal	385	37.3	660	37.1	1,045	37.2	0.898
Postmenopausal	646	62.7	1,119	62.9	1,765	62.8	
Total	1,031	36.7	1,779	63.3	2,810	100	
Biospecimen							
Primary lesion	1,000	73.7	1,382	71.1	2,382	72.1	0.096
Other lesion	357	34.6	563	28.9	920	27.9	
Total	1,357	41.1	1,945	58.9	3,302	100	
Histotype							
Ductal	639	70.8	1,214	74.3	1,853	73	0.175
Lobular	194	21.5	314	19.2	508	20	
Other	69	7.6	107	6.5	176	6.9	
Total	902	35.6	1,635	64.4	2,537	100	
T							
1	509	55.8	807	48.7	1,316	51.2	0.007
2	294	32.2	618	37.3	912	35.5	
3	71	7.8	142	8.6	213	8.3	
4	38	4.2	89	5.4	127	4.9	
Total	912	35.5	1,656	64.5	2,568	100	
*N*							
0	556	58.8	937	55.6	1,493	56.8	0.01
1	272	28.8	464	27.6	736	28	
2	71	7.5	148	8.8	219	8.3	
3	46	4.9	135	8	181	6.9	
Total	945	35.9	1,684	64.1	2,629	100	
ER							
Positive	983	67	1,894	87.1	2,877	79	<0.001
Negative	484	33	280	12.9	764	21	
Total	1,467	40.3	2,174	59.7	3,641	100	
PgR							
Positive	789	54.7	1,542	71.8	2,331	64.9	<0.001
Negative	654	45.3	606	28.2	1260	35.1	
Total	1,443	40.2	2,148	59.8	3,591	100	
G							
1	67	8.8	139	10.6	206	9.9	0.0499
2	272	35.6	514	39.1	786	37.8	
3	426	55.7	660	50.3	1086	52.3	
Total	765	36.8	1,313	63.2	2,078	100	
Ki67							
Median	16		18		18		0.892
IQR	9–30		10–27		10–27		
Min–max	0.5–95		0.5–95		0.5–95		
Pts with available data	433	36.4	756	63.6	1,189	100	
≤14%	190	43.9	294	38.9	484	40.7	0.092
>14%	243	56.1	462	61.1	705	59.3	
<20%	236	54.5	411	54.4	647	54.4	0.963
≥20%	197	45.5	345	45.6	542	45.6	
TILs							
Median	1		1		1		0.218
IQR	0–5		1–5		1–5		
Min–max	0–80		0–80		0–80		
Pts with available data	102	37.2	172	62.8	274	100	
PAM50 subtypes							
Luminal A	193	28.7	459	50.8	652	41.4	<0.001
Luminal B	127	18.9	260	28.8	387	24.6	
HER2-enriched	40	5.9	32	3.5	72	4.6	
Basal-like	294	43.7	120	13.3	414	26.3	
Normal-like	19	2.8	32	3.5	51	3.1	
Total	673	42.7	903	57.3	1,576	100	
HR status							
HR-positive	1,025	69.6	1,937	88.2	2,962	80.8	<0.001
TNBC	448	30.4	258	11.8	706	19.2	
Total	1,473	40.2	2195	59.8	3,668	100	

^a^Chi-square test for differences in proportions, Kruskalis–Wallis and Wilcoxon rank sum test with continuity correction, where appropriate, for continuous variables (median comparisons).

*Pts* patients, *HR* hormone receptors, *IQR* interquartile range, *IHC* immunohistochemical, *TILs* tumor-infiltrating lymphocytes.

### Reproducibility of the HER2-low classification

To evaluate the reproducibility of HER2 IHC scoring among pathologists, we scanned 200 HER2 IHC stained slides from 100 independent cases of the Hospital Clinic case series. The images were representative of the 4 HER2 IHC categories (i.e., 0, 1+, 2+ and 3+). Five breast cancer-specialized pathologists (BG, ES, RF, GP, and VP), coming from four different institutions (Clinic, VHIO, VHV, and Campus Bio-Medico), revised and scored the 100 cases in a blinded fashion. Overall, 35 discordant cases (35%) were observed. The discordances were between IHC 1+ vs. 0 (*n* = 15), 1+ vs. 2+ (*n* = 12), 2+ vs. 0 (*n* = 1), 3+ vs. 1 + (*n* = 1), and 3+ vs. 2+ (*n* = 6) scores. In most cases (25 of 35, 71.4%), only one pathologist was discordant with the others. The multi-rater overall kappa concordance score was 0.79 (*p* < 0.001), which is considered a substantial agreement. The kappa scores according to the HER2 IHC categories 0, 1+, 2+, and 3+ were 0.82 (almost perfect agreement), 0.67 (substantial agreement), 0.74 (substantial agreement) and 0.92 (almost perfect agreement), respectively (*p* < 0.001). Similar results were obtained when the HER2 3+ cases were removed (data not shown).

### Distribution of the PAM50 intrinsic subtypes

**Fig. 3:**
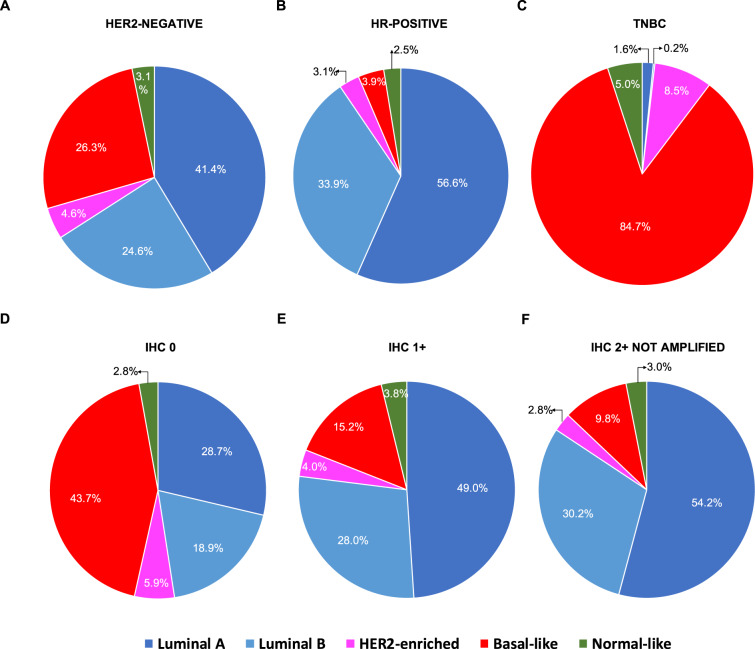
Intrinsic subtype distribution according to HER2 status and HR status. HR hormone receptors, TNBC triple-negative breast cancer, IHC immunohistochemistry, ISH in situ hybridization (including either FISH, SISH, and CISH). Number of patients in **A** (*n* = 1576), **B** (*n* = 1137); **C** (*n* = 437); **D** (*n* = 673); **E** (*n* = 701); **F** (*n* = 325).

PAM50 intrinsic subtypes were available from 1,576 (42.7%) patients. Intrinsic subtypes were differentially distributed among the three IHC-based groups, as well as between HER2-low and HER2 0 tumors (*p* < 0.001 for both) (Fig. 3, Table 1, and Supplementary Table 1). Intrinsic subtypes distribution varied also between HR-positive and TNBC (*p* < 0.001) (Fig. 3 and Supplementary Table 2). Specifically, Luminal A tumors were more frequent within the IHC 2+ (54.2%), HER2-low (50.8%) and HR-positive (56.6%) groups compared to IHC 1+ (49.0%), IHC 0 (28.7%) and TNBC (1.6%). Similarly, Luminal B were more frequent within the IHC 2+ (30.2%), HER2-low (28.8%) and HR-positive (33.9%) groups compared to IHC 1+ (28.0%), IHC 0 (18.9%) and TNBC (0.2%); HER2-enriched (HER2-E) were more frequent within the IHC 0 (5.9%) and TNBC (8.5%) groups compared to IHC 2+ (2.8%), IHC 1+ (4.0%), HER2-low (3.5%) and HR-positive tumors (3.1%); Basal-like tumors were mostly concentrated within the IHC 0 (43.7%) and TNBC (84.7%) groups compared to IHC 2+ (9.8%), IHC 1+ (15.2%), HER2-low (13.4%) and HR-positive tumors (3.9%).

Within HR-positive disease, intrinsic subtypes were differentially distributed between HER2-low and HER2 0 tumors, as well as according to IHC score (*p* < 0.001 in both cases; Table 2 and Supplementary Table 3). Specifically, Luminal B and Basal-like subtypes were less frequent in HER2-low compared to HER2 0 (Luminal B: 8.0% vs. 34.9%; Basal-like: 1.9% vs. 33.4%), while Luminal A subtype was more frequent in HER2-low compared to HER2 0 (58.9% vs. 2.8%). There was no significant difference in subtype distribution in TNBC according to HER2-low status and IHC score (*p* = 0.438 and *p* = 0.284, respectively; Table 2 and Supplementary Table 3). When comparing HR-positive and TNBC according to the same HER2 IHC score, intrinsic subtypes were significantly differentially distributed, with Basal-like tumors being the predominant subtype in each TNBC/HER2 subset (85.2% in HER2 0, 85.4% in HER2 1+, 78.4% in HER2 2+). As expected, Luminal A (51.8% in HER2 0, 57.9% in HER2 1+, 60.6% in HER2 2+), followed by Luminal B subtype (34.9% in HER2 0, 33.1% in HER2 1+, 33.8% in HER2 2+), were the most frequent in each HR-positive/HER2 subset (Supplementary Table 4).

**Table 2:** PAM50 intrinsic subtypes distribution within HR-positive and TN tumors according to HER2 status.

HR-positive	HER2 0		HER2-low		Overall		*p*^a^
	*N*	%	*N*	%	*N*	%	
PAM50 subtypes							
Luminal A	187	51.8	457	58.9	644	56.6	<0.001
Luminal B	126	34.9	259	33.4	385	33.9	
HER2-enriched	12	3.3	23	3.0	35	3.1	
Basal-like	29	8.0	15	1.9	44	3.9	
Normal-like	7	1.9	22	2.8	29	2.6	
Total	361	31.8	776	100.0	1,137	100.0	
TNBC							
PAM50 subtypes							
Luminal A	5	1.6	2	1.6	7	1.6	0.438
Luminal B	1	0.3	0	0.0	1	0.2	
HER2-enriched	28	9.0	9	7.1	37	8.5	
Basal-like	265	85.2	105	83.3	370	84.7	
Normal-like	12	3.9	10	7.9	22	5.0	
Total	311	71.2	126	100.0	437	100.0	

*HR* hormone receptor, *TNBC* triple-negative breast cancer.

^a^Chi-square test for differences in proportions.

Finally, we investigated if the distribution of PAM50 subtypes within HER2-low breast cancer differed according to *ERBB2* mRNA levels. To approach it, we divided all patients with HER2-negative disease into tertiles (i.e., from low to high: T1, T2, and T3) based on *ERBB2* expression (Table 3). As expected, subtype distribution differed in HER2-low breast cancer according to *ERBB2* levels (*p* < 0.001) with the T2-3 group being more enriched with Luminal A, Luminal B and HER2-E subtypes (51.5%, 34.9%, and 6.3%) compared to the T1 group (31.7%, 15.8%, and 3.6%). On the contrary, the Basal-like subtype was more frequent in the T1 group compared to the T2-3 group (44.6% vs 2.9%). The results were similar when comparing either *ERBB2* high/HER2-low and *ERBB2* low/HER2-low tumors with the whole HER2-low population (*p* < 0.001 both) (Table 3).

**Table 3:** Intrinsic subtypes distribution in HER2-low tumors according to *ERBB2* mRNA levels.

Intrinsic subtype	*ERBB2* high (T3-T2)		*ERBB2* low (T1)		HER2-low		*p*^a^	*p*^b^	*p*^c^
	*N*	%	*N*	%	*N*	%			
Luminal A	140	51.5	44	31.7	184	44.8	<0.001	<0.001	<0.001
Luminal B	95	34.9	22	15.8	117	28.5			
HER2-enriched	17	6.3	5	3.6	22	5.4			
Basal-like	8	2.9	62	44.6	70	17.0			
Normal-like	12	4.4	6	4.3	18	4.4			
Total	272	66.2	139	33.8	411	100.0			

*ERBB2* is italicized, as per standard gene ID formatting guidelines. T1**:** tertile one; T2**:** tertile two; T3**:** tertile 3.

^a^Referred to the comparison between ERBB2 high vs. low.

^b^Referred to ERBB2 high vs. the overall HER2-low population.

^c^Referred to ERBB2 low vs. the overall HER2-low population.

### PAM50 and individual gene expression analyses

PAM50 and individual gene expression data was available in 1,320 (35.8%) patients. The full list of genes and subtypes’ signatures evaluated for differential expression analyses in the overall HER2-negative population and according to HR status are reported in Supplementary Table 5.

In the overall population, 34 of 55 genes (61.8%) were found differentially expressed between HER2-low and HER2 0 (false-discovery rate [FDR] < 5%) (Table 4, Supplementary Table 6 and Supplementary Fig. 1). Specifically, 14 genes (41.2%) were found significantly downregulated in HER2-low compared to HER2 0, including proliferation-related genes (e.g., *CCNB1, CCNE1, MELK, MKI67, MYBL2* etc.), Basal-like-related genes (e.g., *KRT14, KRT17, KRT5, FOXC1, MYC* etc.), tyrosine-kinase receptors (i.e., *EGFR, FGFR4*), and three PAM50 signatures (i.e., HER2-E, Basal-like and Normal-like). Conversely, 20 genes (58.8%) were found significantly upregulated in HER2-low compared to HER2 0, including luminal-related genes (e.g., *BCL2, BAG1, FOXA1, ESR1, PGR, GPR160* and *AR*) and two PAM50 signatures (i.e., Luminal A and B). According to HR status, similar findings were observed in HR-positive disease as in the general population (Table 4, Supplementary Table 6, and Supplementary Fig. 2). In TNBC, however, no individual gene, or PAM50 signature, was found differentially expressed between HER2-low and HER2 0. Similar findings were observed when HER2-low disease was subdivided into 1+ and 2+ (Table 4, Supplementary Table 6, and Supplementary Fig. 3).

**Table 4:** Top 20 differentially expressed genes between HER2-low and HER2 0 disease.

Gene symbol	Association	Overall		HR-positive		TNBC	
		Score(d) (strength of relationship)	FDR^a^	Score(d) (strength of relationship)	FDR^a^	Score(d) (strength of relationship)	FDR^a^
*ESR1*	Higher in HER2-low	14.3	0	5.0	0	1.0	100
*FOXA1*	Higher in HER2-low	13.3	0	4.9	0	1.0	100
*NAT1*	Higher in HER2-low	12.3	0	4.1	0	−0.3	68.3
*SLC39A6*	Higher in HER2-low	11.6	0	4.0	0	−0.4	64.3
*PGR*	Higher in HER2-low	11.2	0	3.2	0	0.5	100
*AR*	Higher in HER2-low	10.6	0	—	—	0.4	100
*ERBB2*	Higher in HER2-low	10.0	0	5.2	0	1.7	100
*MAPT*	Higher in HER2-low	9.9	0	2.9	0	−0.2	68.3
*MLPH*	Higher in HER2-low	8.8	0	2.6	0	0.0	68.3
*BCL2*	Higher in HER2-low	8.2	0	2.5	0	0.0	68.3
*CENPF*	Lower in HER2-low	−7.0	0	−1.8	0	−1.0	64.3
*EXO1*	Lower in HER2-low	−7.1	0	−2.4	0	−1.2	64.3
*ANLN*	Lower in HER2-low	−7.4	0	−2.3	0	−0.4	64.3
*ORC6L*	Lower in HER2-low	−7.6	0	−2.3	0	−0.8	64.3
*KNTC2*	Lower in HER2-low	−7.8	0	−2.3	0	−0.7	64.3
*CEP55*	Lower in HER2-low	−7.8	0	−1.3	3.2	−1.1	64.3
*PHGDH*	Lower in HER2-low	−8.4	0	−1.7	0	−1.2	64.3
*FOXC1*	Lower in HER2-low	−8.4	0	−0.7	9.7	0.2	100
*MKI67*	Lower in HER2-low	−8.7	0	−2.4	0	−1.0	64.3
*CCNE1*	Lower in HER2-low	−9.6	0	−3.0	0	−0.9	64.3

In the table only significantly subtype signatures, top-10 upregulated and top-10 downregulated genes for the overall population are reported, along with their corresponding result in the HR + and TNBC populations. Genes are italicized, as per standard formatting guidelines.

*HR* hormone receptors, *TNBC* triple-negative breast cancer, FDR false-discovery rate.

^a^Significant if FDR < 5.0; Score(d): a *T*-statistic value that reflects a standardized change in expression and measures the strength of the relationship between gene expression and the HER2-low category (vs. HER2 0).

### Gene expression profiles according to HER2 expression and HR status

**Fig. 4:**
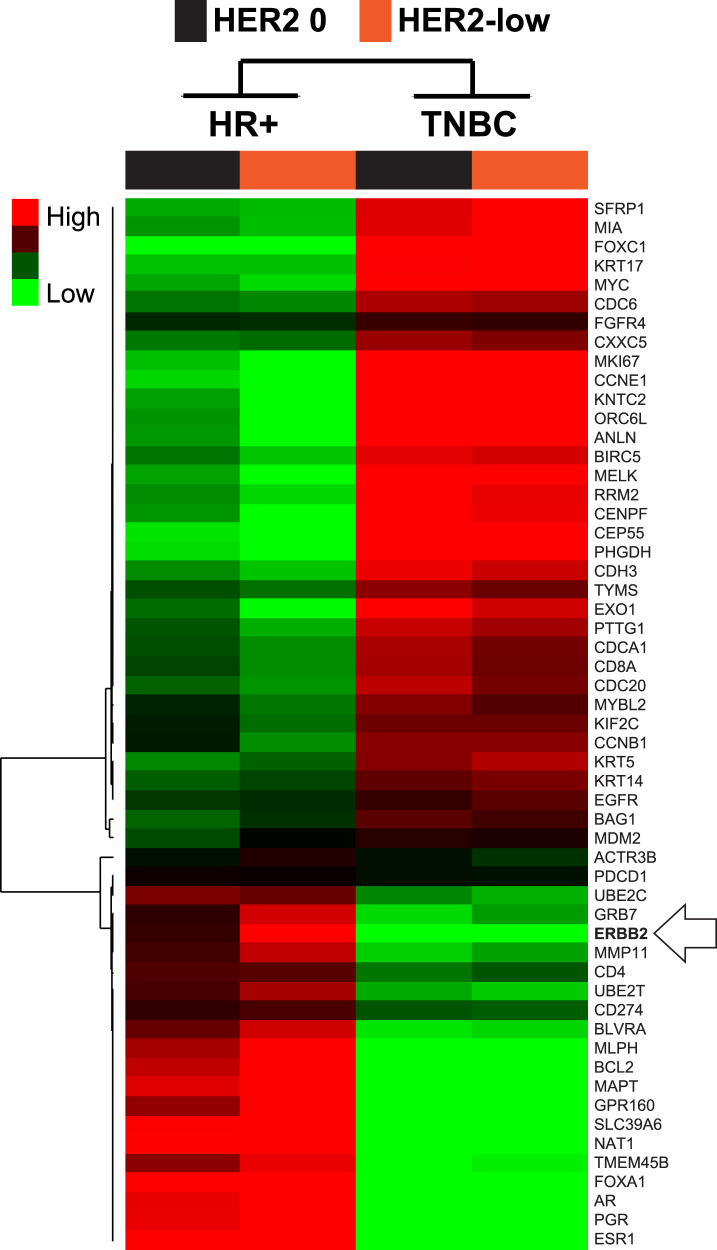
Gene expression profiles of HER2-negative breast cancer according to HER2 expression and HR status. Supervised clustering of 55 genes across four tumor classes defined according to HER2 IHC expression and HR status. All samples and gene expression data in each category have been combined into a single group. For each gene in a group, we calculated the standardized mean difference between the gene’s expression in that class vs. its overall mean expression in the dataset using a 4-class Significance Analyses of Microarrays. The red color represents relative high gene score, green represents relative low gene score, and black represents median gene score. HR-positive hormone receptor positive, TNBC triple-negative breast cancer.

The previous results suggested that HR status is a key determinant of the underlying biology of HER2-low breast cancer. To further explore this, we evaluated the overall gene expression profile of HER2-negative breast cancer according to HER2 expression (i.e., HER2 0, 1+ and 2+) and HR status (i.e., positive and negative). The result clearly shows that HR status is the main driver of the underlying biology (Fig. 4 and Supplementary Table 7). As expected, proliferation-related genes (e.g., *CCNE1, MKI67* and *EXO1*) were found more expressed in TNBC compared to HR-positive, regardless of HER2 IHC status (i.e., HER2-low vs. HER2 0). On the contrary, luminal-related genes (e.g., *ESR1, AR*, and *BCL2*) and *ERBB2* were found more expressed in HR-positive compared to TNBC, regardless of HER2 IHC status. Of note, the highest *ERBB2* expression was found in the HR-positive/HER2-low group. Finally, concordant with the previous results, HER2-low tumors within HR-positive disease showed a relatively lower expression of proliferation-related genes and higher expression of luminal-related genes compared to the HER2 0 group (Supplementary Fig. 4 and Supplementary Table 8).

### ERBB2 expression analysis

**Fig. 5:**
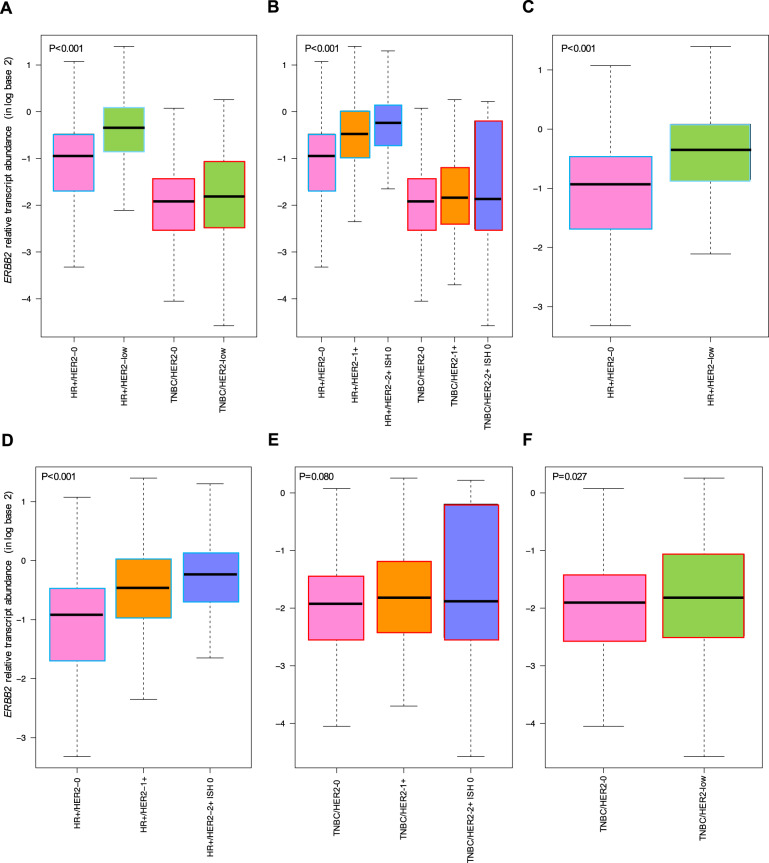
*ERBB2* mRNA levels within the overall, HR-positive and TNBC populations according to HER2-low expression. Relative transcript abundance of *ERBB2* (HER2 gene) within the overall population (*n* = 871) and within HR-positive disease (*n* = 494) and TNBC (*n* = 377) according to HER2 IHC-based expression. The boxes represent the interquartile range (25th and 75th percentiles), and the horizontal line in the box represents the median value. The whiskers show the range of largest and smallest values. HR-positive hormone receptor positive, TNBC triple-negative breast cancer.

The previous observation that *ERBB2* levels differ according to HER2 IHC expression (HER2 0, 1+, and 2+) and HR status was somewhat unexpected. To further explore this finding, we formally compared the abundance of *ERBB2* in HR-positive disease and TNBC based on HER2 IHC expression. *ERBB2* levels were statistically significantly higher in HR-positive tumors compared to TNBC regardless of HER2 IHC expression (*p* < 0.001; Fig. 5A, B). Within HR-positive disease, *ERBB2* levels were significantly higher in HER2-low tumors compared to HER2 0 (1.4-fold mean difference, *p* < 0.001, Fig. 5C), with the highest amount observed in HER2 IHC 2+ tumors, followed by 1+ and 0 (Fig. 5D), in decreasing order (1.7-fold mean difference between HER2 2+ vs. HER2 0). Within TNBC, there was no statistically significantly difference in *ERBB2* levels across the three HER2 IHC groups (*p* = 0.080, Fig. 5E); however, TNBC/HER2-low tumors showed statistically significantly higher levels of *ERBB2* compared to HER2 0 tumors (*p* = 0.027), although the absolute mean difference was very small (Fig. 5F).

### Prognosis of HER2-low in advanced HER2-negative breast cancer

**Fig. 6:**
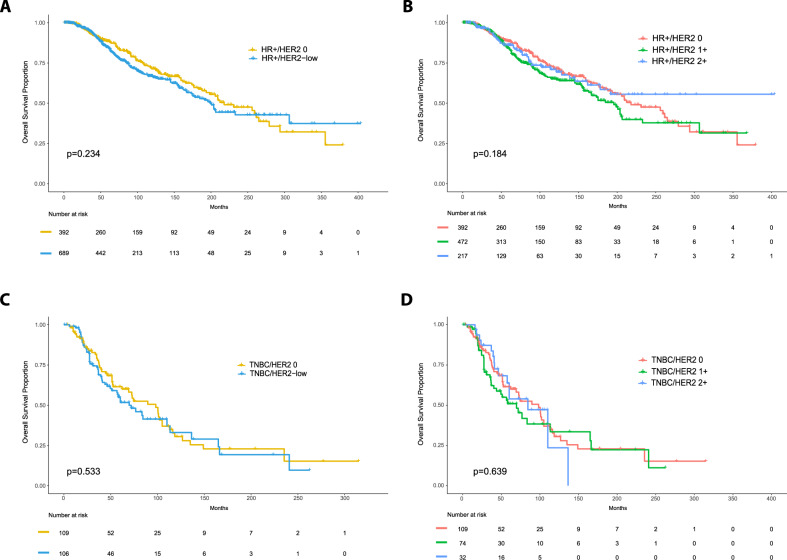
Overall survival in patients with advanced HER2-negative breast cancer according to HER2 expression. The figure shows Kaplan–Meier curves of overall survival for HER2-low vs HER2 0 tumors in the HR-positive (**A**) and TNBC (**C**) populations, as well as OS curves for HER2 2+ vs. HER2 1+ vs. HER2 0 tumors for the HR-positive (**B**) and TNBC (**D**) populations with number at risk shown at the bottom of each box. *p*-values for log-rank tests are also reported; HR-positive hormone receptor positive, TNBC triple-negative.

We conducted an exploratory overall survival (OS) analysis in 1,304 patients with advanced breast cancer across two datasets (i.e., Memorial Sloan Kettering Cancer Center database^18^ and Hospital Clinic internal database). OS was defined from the date of the first diagnosis of breast cancer. The median follow-up for the overall population was 90.3 months (95% confidence interval [CI]: 84.6–99.4). In all patients, no statistically significantly differences in OS were observed between the HER2-low and HER2 0 groups (*p* = 0.787). Similar results were obtained according to HR status and HER2 IHC levels (Fig. 6).

## Discussion

Our results provide preliminary insights of the clinical and molecular characteristics of HER2-low breast cancer. According to our results, patients with HER2-low disease represent the vast majority (59.7%) of patients with HER2-negative tumors. Clinically, HER2-low breast cancer is apparently more frequent in older and male patients and shows more axillary lymph-node involvement compared to HER2 0 disease. Importantly, we observed that HR status has an important role in HER2-low disease. For example, the frequency of HER2-low disease is higher in HR-positive breast cancer than TNBC (65.4% vs. 36.6%) and most HER2-low tumors are HR-positive (88.2%) or Luminal A or B (79.6%). Another important result of our study is that the vast majority (67.6%) of HER2-low tumors have an IHC 1+ score, regardless of HR status. Interestingly, when HR-positive disease and TNBC are divided according to the HER2 IHC score, no significant difference in subtype distribution is observed in TNBC, which was characterized by a high prevalence of the Basal-like subtype (84.7%), followed by the HER2-E (8.5%) subtype. On the contrary, HR-positive/HER2-low tumors appeared to be characterized by a higher proportion of luminal subtypes compared to HER2 0 tumors. Of note, the HER2-E subtype was infrequent and similarly distributed in HER2-low and HER2 0 breast cancer.

As expected, the differences in subtype distribution according to HER2 IHC expression and HR status are consistent with the observed changes in expression of individual genes. For example, the vast majority of proliferation-related genes and tyrosine-kinase receptor genes are found more expressed in HER2 0 tumors compared to HER2-low tumors, while HER2-low tumors have more expression of luminal-related genes. This finding is especially relevant in HR-positive disease. On the contrary, no clear biological differences are observed in TNBC according to HER2 IHC expression. Overall, these findings suggest that HR-positive/HER2-low tumors are a more distinct biological entity compared to TNBC/HER2-low tumors.

The lack of enrichment of the HER2-E subtype within HER2-low disease is intriguing and somewhat unexpected. However, previous studies have shown that the HER2-E phenotype is not defined by the expression of a single gene such as *ERBB2*. In fact, we and others have previously shown that the two variables (i.e., HER2-E subtype and *ERBB2* levels) provide independent predictive and prognostic information^19^. Overall, this finding clearly highlights the need to separate expression of single genes or receptors from the underlying tumor phenotype.

Recent studies have opened up a new therapeutic scenario by showing potent activity of HER2-targeted novel ADCs in HER2-low breast cancer^8^. To date, T-DXd, a trastuzumab conjugated to eight molecules of deruxtecan, a topoisomerase I inhibitor, is at the most advanced in clinical development. A recently published phase Ib study enrolling highly pretreated patients with advanced HER2-expressing/mutated solid tumors, including HER2-low breast cancer, revealed a remarkable overall response rate (ORR) of 37.0% (95% CI: 24.3–51.3%) in HER2-low breast cancer and an impressive median duration of response of 10.4 months (95% CI: 8.8 month—not evaluable), with no apparent differences in ORR between 1+ and 2+ IHC tumors (35.7% vs. 38.5%)^9^. Interestingly, the ORR did seem to differ according to HR status (40.4% in HR-positive disease and 14.3% in TNBC). This result is concordant with our findings that *ERBB2* levels are more expresses in HR-positive/HER2-low tumors than in TNBC/HER2-low tumors. A phase III trial specifically enrolling patients with HER2-low metastatic breast cancer (i.e., NCT03734029/DESTINY-Breast04) is ongoing. Importantly, we previously demonstrated in HER2-positive disease that *ERBB2* mRNA levels might provide a better selection of patients that benefit to the ADC T-DM1^20^. This might also be the case for HER2-low tumors and might be worth focusing on this aspect in further studies.

SYD985 is another ADC comprises trastuzumab covalently bound to a linker drug containing duocarmycin. This drug also showed a promising ORR of 28 and 40% in HR-positive/HER2-low and TNBC/HER2-low, respectively^21^. In addition, other anti-HER2 ADCs (i.e., PF-06804103, MEDI4276, and XMT-1522) have shown promising activity in HER2-low tumors in the preclinical setting^8,22^, and phase 1 clinical trials are ongoing (clinicaltrials.gov identifier: NCT03284723, NCT02564900, and NCT02952729, respectively).

Tumors with high *ERBB2* mRNA levels, but overall HER2-negative, might also benefit from novel tumor vaccines targeted against the HER2 protein, as shown by a recent randomized phase II trial of HER2-targeted vaccine nelipepimut-S combined with trastuzumab as adjuvant treatment in HER2-low high-risk breast cancer^23^. In this direction, we observed higher levels of TILs in the HER2 2+ group compared to the HER2 0 and 1+ groups, although this analysis was based on a very restricted number of cases. Further studies are needed to study the immune compartment of HER2-low breast cancer.

Our study presents limitations that need attention. First, we retrospectively combined patients from databases pertaining to different studies, with different original purposes and inclusion/exclusion criteria; therefore, patients were not consecutively enrolled and a large proportion of them had metastatic disease. These might explain some of the imbalances that we observed between groups. Additionally, HER2 IHC status was not evaluated centrally; thus, inter-pathologist variability might have affected the results. Moreover, criteria for defining negative or equivocal *ERBB2* amplification have changed over time^1,2^ and most *ERBB2* amplification results were only available in qualitative form (i.e., amplified, not amplified or equivocal). Another limitation is that we did not address intra-tumor HER2 heterogeneity, which represents 1%–34% of all breast tumors^24^ and has clinical and prognostic implications, with poor response to anti-HER2-based regimens and worse prognosis, compared to HER2-positive tumors^24^. However, this feature is more common in HER2 equivocal disease^24^, a condition that was an exclusion criteria in our study, somewhat mitigating this issue. Finally, we limited our genomic analysis to the PAM50 genes and five additional genes. Thus, broader genomic analyses are likely to shed more light on this topic.

To our knowledge, this is the first comprehensive study focused specifically on HER2-low breast tumors. We provided extensive comparisons among the three different IHC-based classes of HER2-negative breast cancer and according to HR status. We found that HER2-low breast tumors are complex and heterogeneous, with no specific prognostic implications and HR-positive/HER2-low emerge as a more distinct biological entity compared to the other groups. In addition, the evidence of *ERBB2* levels being higher in HER2-low/HER2 2+ tumors (especially in the HR-positive) compared to HER2 1+ /0 is in line with some previous findings from single institutions-based studies, and contributes to reassure about the reliability of our results^25,26^. Similarly, the high prevalence of luminal disease in HER2-low disease has also been observed in other studies^24^. Finally, the concordance analysis of HER2 scoring by different pathologists showed an almost perfect agreement for HER2 0 and 3+ scores; however, the agreement for the HER2 1+ and 2+ categories was only substantial, according to Landis and Koch interpretation^27^. This result clearly suggests that more efforts are needed to standardize the scoring of HER2-low disease and potentially implement new and more sensitive assays that can help better discriminate HER2 levels within HER2-negative breast cancer.

## Methods

### Patients datasets

All non-overlapping publicly available breast datasets (i.e., 12 studies and 6477 patients) were interrogated from the cBio Cancer Genomics Portal (http://cbioportal.org). From these databases, HER2-negative tumors with known IHC and HER2 amplification status were extracted^10–13^. Other patients were extracted from internal databases from the Hospital Clinic (Barcelona, Spain), from two SOLTI clinical trials (SOLTI 1501-VENTANA and SOLTI 1402-CORALEEN)^14,15^, from the Spanish Cancer Research Group (GEICAM)/CIBOMA study^16^ and from a previously published collaboration between Hospital Clinic (Barcelona, Spain), Hospital Vall d’Hebron (Barcelona, Spain), University Campus Bio-Medico (Roma, Italy) and GEICAM^17^ (see Supplementary Table 9 for study details). All studies had received proper ethical approval by the local institutional research ethics committee of all participating institutions and patients had given their consent to participate.

### Inclusion criteria

Patients were included if they were HER2-negative with known IHC and HER2 amplification status and if they had at least one of the following information available: (1) clinicopathological features, (2) PAM50 gene expression data, and (3) PAM50 intrinsic subtype identified. The following clinical-pathological features were evaluated, when available: Ki67 IHC, histological grade, estrogen receptor and progesterone receptor status, age at diagnosis, menopausal status, tumor sample origin (primary vs. metastatic), histological subtype and TILs.

### IHC-based classification

Tumors were divided into HR-positive (i.e., ER and/or PgR ≥1%) or TNBC, defined as ER < 1% and PgR<1%. In addition, tumors were classified into HER2 0, in case of an IHC score of 0, and HER2-low, defined as HER2 IHC of 1+ or 2+ with an HER2 amplification negative result by in situ hybridization (ISH) techniques. HER2 IHC 0 and 1+ were considered HER2 0 and HER2-low, respectively, unless ISH-based data was available and reported as HER2-amplified. HER2 status in each cohort had been previously determined using standard FDA-approved antibodies and ISH-techniques and classified according to the ASCO/CAP guidelines^1,2^. Whenever available, we interpreted ISH-derived HER2/CEP17 ratio value and *ERBB2* copy number results jointly with HER2 IHC score, according to last ASCO/CAP guidelines^1^. More specifically, tumors with an average HER2 copy number <4.0 signals/cell, were considered HER2-negative, and also HER2-low in case of an IHC score of 1+ or 2+, irrespective of the HER2/CEP17 ratio. However, if the HER2/CEP17 ratio was ≥2.0 and HER2 IHC 3+, tumors were considered HER2-positive and excluded^1^.

In case of available average HER2 copy number ≥4.0 and <6.0 signals/cell without HER2/CEP ratio and an IHC 3+, the tumor was considered positive and excluded. In case of IHC 0 or 1+, the tumor was considered HER2-negative, and also HER2-low in the latter case^1^. In case of IHC 2+, considering the unfeasibility of a retesting, in our case, if the categorization HER2-positive/negative was available from the original dataset, it was adopted and the tumor was considered HER2-negative and HER2-low. If the categorization was not provided, the sample was excluded.

In case of IHC score 0, 1+ or 2+, and a concurrent average HER2 copy number ≥4.0 and <6.0 signals/cell, with HER2/CEP17 ratio <2.0, the tumor was considered HER2-negative, and HER2-low in the last two cases. On the contrary, if the HER2/CEP17 watio was ≥2.0, the tumor was considered HER2-positive and excluded^1^.

In case of HER2 copy number ≥6.0 signals/cell, the tumor was considered HER2-positive and excluded in case of IHC of 2+ or 3+, regardless of the HER2/CEP17 ratio result, but in case of HER2/CEP17 ratio <2.0 and IHC 0 or 1+, the tumor was considered negative, and also HER2-low in the second case^1^.

Patients with a persistent HER2 equivocal result were excluded^1^.

To evaluate the concordance of the HER2 IHC categories among pathologists, we performed an inter-pathologist concordance analysis across 100 independent cases of HER2 staining (HER2 0, 1+, 2+, and 3+). Five independent breast cancer-specialized pathologists (i.e., BG, ES, RF, VP, and GP) from four institutions (i.e., Hospital Clinic, VHIO, HVH, and Campus Bio-Medico) were involved. Blinded scores were provided to FS and AP, who performed the concordance analysis.

### PAM50 subtypes and gene expression data

We obtained PAM50 subtype information and individual gene expression data from 9 of the 13 retrospective cohorts (Hospital Clinic internal series, SOLTI and GEICAM trials reported in Supplementary Table 9). An nCounter-based research version of PAM50 had been previously used^28,29^. Intrinsic subtypes and raw gene expression data had been obtained from formalin-fixed paraffin-embedded (FFPE) tumor samples. For RNA purification (Roche High Pure FFPET RNA isolation kit), at least 1 to 3 10-μm FFPE slides had been used for each tumor specimen, and macrodissection performed, when needed, to avoid normal breast tissue contamination. A minimum of ~150 ng of total RNA had been used to measure the expression of 50 breast cancer-related genes, 4 immune-related genes, androgen receptor gene (full gene list included in Supplementary Table 5), and 5 housekeeping genes (*ACTB*, *MRPL19*, *PSMC4*, *RPLP0*, and *SF3A1*) using the nCounter platform (NanoString Technologies, Seattle WA)^28,30^. Data had been log base 2 transformed and normalized using the five housekeeping genes. Intrinsic subtyping (Luminal A, Luminal B, HER2-E, Basal-like and Normal-like) had been previously performed using the research-based PAM50 intrinsic subtype predictor^29^. We also retrieved intrinsic subtypes from the publicly available TCGA database (see “Data availability” section for further information).

### Statistical analysis

Patient and tumor characteristics were analyzed using chi-square (*χ*^2^) test, Fisher’s exact test, Kruskalis–Wallis and Wilcoxon rank sum test with continuity correction, where appropriate. The concordance analysis among pathologists was performed using the Fleiss’ Kappa. The agreement among pathologists was considered poor for *k* < 0, low for *k* = 0.01–0.20, fair for *k* = 0.21–0.40, moderate for *k* = 0.41–0.60, substantial for *k* = 0.61–0.80, and almost perfect for *k* = 0.81–1.00^27^.

All differences were considered significant at *p* < 0.05. Bonferroni–Holm method was used to control the family-wise error rate in case of multiple comparisons.

OS was evaluated for patients with homogeneous follow-up with available or computable survival data. Such patients pertained to the Memorial Sloan Kettering Cancer Center (MSKCC)’s subset of the cBio Cancer Genomics Portal group and to the Hospital Clinic of Barcelona subset. All patients were affected by metastatic disease and presented available information regarding primary tumor diagnosis.

The OS distributions were estimated using the Kaplan–Meier method and the log-rank test was used to assess the difference in survival distribution between the groups^31^. Censoring was done at the date of last available follow-up. Significance Analysis of Microarray (SAM) for unpaired samples (multiclass and two class) was used to compare gene expression profiles between groups^32^. Differences were considered significant at an FDR < 5%. All analyses were performed with R version 3.6.1^33^, Cluster 3.0, Javatreeview 1.1.6r4^34^ and Microsoft Excel.

### Reporting summary

Further information on research design is available in the Nature Research Reporting Summary linked to this article.

## Supplementary information

Supplementary materials

Dataset 1

Reporting Summary

## Data Availability

This study involved the collection and analysis of clinicopathological and PAM50 gene expression data from multiple publicly available datasets^35–46^. The following cBioPortal datasets were used: https://identifiers.org/cbioportal:breast_msk_2018; https://identifiers.org/cbioportal:bfn_duke_nus_2015; https://identifiers.org/cbioportal:brca_mskcc_2019; https://identifiers.org/cbioportal:brca_bccrc_xenograft_2014; https://identifiers.org/cbioportal:brca_bccrc; https://identifiers.org/cbioportal:brca_broad; https://identifiers.org/cbioportal:brca_sanger; https://identifiers.org/cbioportal:brca_tcga; https://identifiers.org/cbioportal:brca_igr_2015; https://identifiers.org/cbioportal:brca_metabric; https://identifiers.org/cbioportal:brca_mbcproject_wagle_2017; https://identifiers.org/cbioportal:acbc_mskcc_2015. Data from the internal studies of the Hospital Clinic of Barcelona, and data from patients involved in the SOLTI and GEICAM trials included, are not publicly available to protect patient privacy, but will be made available on reasonable request from the corresponding author, Prof. Aleix Prat (email address: alprat@clinic.cat). An anonymized data file containing all PAM50 normalized gene expression data used for the genomic analyses of this study, is publicly available in the figshare repository^47^, with 10.6084/m9.figshare.13171655. The complete version of the data file used and/or analyzed during the current study, is available upon reasonable request from the corresponding author, as described in the figshare data record above.

## Code availability

R codes are available from the corresponding author on reasonable request.
