# Author Correction: Clinical, pathological, and PAM50 gene expression features of HER2-low breast cancer

**DOI:** 10.1038/s41523-023-00538-x

**Published:** 2023-04-29

**Authors:** Francesco Schettini, Nuria Chic, Fara Brasó-Maristany, Laia Paré, Tomás Pascual, Benedetta Conte, Olga Martínez-Sáez, Barbara Adamo, Maria Vidal, Esther Barnadas, Aranzazu Fernández-Martinez, Blanca González-Farre, Esther Sanfeliu, Juan Miguel Cejalvo, Giuseppe Perrone, Giovanna Sabarese, Francesca Zalfa, Vicente Peg, Roberta Fasani, Patricia Villagrasa, Joaquín Gavilá, Carlos H. Barrios, Ana Lluch, Miguel Martín, Mariavittoria Locci, Sabino De Placido, Aleix Prat

**Affiliations:** 1 Department of Clinical Medicine and Surgery, University of Naples Federico II, Naples, Italy; 2 Translational Genomics and Targeted Therapeutics in Solid Tumors, August Pi i Sunyer Biomedical Research Institute, Barcelona, Spain; 3 SOLTI Breast Cancer Research Group, Barcelona, Spain; 4 Department of Medical Oncology, Hospital Clínic, Barcelona, Spain; 5 Department of Genetics, University of North Carolina, Chapel Hill, NC USA; 6 Department of Medical Oncology, Ospedale Policlinico San Martino, University of Genova, Genova, Italy; 7 Department of Pathology, Hospital Clínic, Barcelona, Spain; 8 INCLIVA Biomedical Research Institute, Hospital Clínico Universitario Valencia, University of Valencia, Valencia, Spain; 9 Pathology Department, Campus Bio-Medico University, Rome, Italy; 10 Vall d´Hebron University Hospital, Barcelona, Spain; 11 GEICAM, Grupo Español de Investigación en Cáncer de Mama, Madrid, Spain; 12 Vall d’Hebron Institute of Oncology (VHIO), Barcelona, Spain; 13 Instituto Valenciano de Oncología (IVO), Valencia, Spain; 14 Centro de Pesquisa Clínica Hospital São Lucas da PUCRS, Porto Alegre, Brazil; 15 LACOG, Latin American Cooperative Oncology Group, Porto Alegre, Brazil; 16 Hospital Universitario Clínico Valencia, Valencia, Spain; 17 Biomedical Research Centre Network in Cancer (CIBERONC), Valencia, Spain; 18 Hospital Gregorio Marañon, Madrid, Spain; 19 Department of Neuroscience, Reproductive Sciences and Dentistry, University of Naples Federico II, Naples, Italy

**Keywords:** Breast cancer, Breast cancer, Translational research, Cancer genomics, Breast cancer

Correction to: *npj Breast Cancer* 10.1038/s41523-020-00208-2, published online 04 January 2021

The original version of the Acknowledgements contained an error in the acknowledgement of the funding source. The correct version of the Acknowledgements is:

The RESCUER project has received funding from the European Union’s Horizon 2020 Research and Innovation Programme under Grant agreement No. 847912 (to A.P.), the Instituto de Salud Carlos III-PI16/00904 (to A.P.), Pas a Pas (to A.P.), Save the Mama (to A.P.), Breast Cancer Now-2018NOVPCC1294 (to A.P.), Fundación Científica Asociación Española Contra el Cáncer-Ayuda Postdoctoral AECC 2017 (to F.B.-M.), Fundación SEOM, Becas FSEOM para Formación en Investigación en Centros de Referencia en el Extranjero 2018 (to T.P.) and PhD4MD - Departament de Salut expedient SLT008/18/00122 (to N.C.).

This replaces the previous incorrect version of the Acknowledgements:

This work was supported by the grants from the European Union’s Horizon 2020 Research and Innovation Programme under Grant agreement No. 847912 (to A.P.), the Instituto de Salud Carlos III-PI16/00904 (to A.P.), Pas a Pas (to A.P.), Save the Mama (to A.P.), Breast Cancer Now-2018NOVPCC1294 (to A.P.), Fundación Científica Asociación Española Contra el Cáncer-Ayuda Postdoctoral AECC 2017 (to F.B.-M.), Fundación SEOM, Becas FSEOM para Formación en Investigación en Centros de Referencia en el Extranjero 2018 (to T.P.) and PhD4MD - Departament de Salut expedient SLT008/18/00122 (to N.C.).

This has been corrected in both the PDF and HTML versions of the Article.

