# Supplementary material for: Clinical, pathological, and PAM50 gene expression features of HER2-low breast cancer
Source: NPJ Breast Cancer. 2021 Jan 4;7:1. doi: 10.1038/s41523-020-00208-2 (PMC7782714; doi:10.1038/s41523-020-00208-2)
Supplement: Supplementary file 1 — Supplementary materials [file 41523_2020_208_MOESM1_ESM.pdf]

Supplementary tables

Supplementary table 1. Demographics according to HER2 IHC status

DEMOGRAPHICS	HER2-NEGATIVE								P*
	HER2 0		HER2 1+		HER2 2+ NON-AMPLIFIED		OVERALL POPULATION		
	N	%	N	%	N	%	N	%	
	1486	40.3	1489	40.4	714	19.3	3689	100	
**Age at diagnosis (years)**									
Median	55		58		60		58		**0.005**
IQR	46 - 65		48 - 67		49 - 68		48 - 67		
Min - max	24 - 93		26 - 96		26 - 90		24 - 96		
Pts with available data	259	27.4	427	45.2	258	27.3	944	100	
**Sex**									
Male	0	0	13	0.9	2	0.3	15	0.4	**0.001**
Female	1486	100	1475	99.1	712	99.7	3673	99.6	
Total	1486	40.3	1488	40.4	714	19.3	3688	100	
**Menopaual status**									
Pre/perimenopausal	385	37.3	454	37.4	206	36.4	1045	37.2	0.908
Postmenopausal	646	62.7	759	62.6	360	63.6	1765	62.8	
Total	1031	36.7	1213	43.2	566	20.1	2810	100	
**Biospecimen**									
Primary lesion	1000	73.7	959	79.1	423	74.7	2382	72.1	0.143
Other lesion	357	34.6	377	28.7	186	32.9	920	27.9	
Total	1357	41.1	1336	40.5	609	18.4	3302	100	
**Histotype**									
Ductal	639	70.8	799	73	415	76.9	1853	73	0.18
Lobular	194	21.5	221	20.2	93	17.2	508	20	
Other	69	7.6	75	6.8	32	5.9	176	6.9	
Total	902	35.6	1095	43.2	540	21.3	2537	100	
**T**									
1	509	55.8	573	50.7	234	44.5	1316	51.2	**0.005**
2	294	32.2	409	36.2	209	39.7	912	35.5	
3	71	7.8	93	8.2	49	9.3	213	8.3	
4	38	4.2	55	4.9	34	6.5	127	4.9	
Total	912	35.5	1130	44	526	20.5	2568	100	
**N**									
0	556	58.8	662	57.3	275	52	1493	56.8	**0.009**
1	272	28.8	300	26	164	31	736	28	
2	71	7.5	104	9	44	8.3	219	8.3	
3	46	4.9	89	7.7	46	8.7	181	6.9	
Total	945	35.9	1155	43.9	529	20.1	2629	100	
**Metastatic status**									

Metastatic Yes	529	65.6	601	61.3	280	64.1	1410	63.4	0.173
Metastatic No	278	34.4	379	38.7	157	35.9	814	36.6	
Total	807	36.3	980	44.1	437	19.6	2224	100	
Ab initio Yes	136	10	151	11.4	80	13.4	367	11.2	0.087
Ab initio No	1218	90	1172	88.6	515	86.6	2905	88.8	
Total	1354	41.4	1323	40.4	595	18.2	3272	100	
**ER**									
Positive	983	67	1261	85.9	633	89.7	2877	79	**<0.001**
Negative	484	33	207	14.1	73	10.3	764	21	
Total	1467	40.3	1468	40.4	706	19.3	3641	100	
**PgR**									
Positive	789	54.7	1030	70.9	512	73.7	2331	64.9	**<0.001**
Negative	654	45.3	423	29.1	183	26.3	1260	35.1	
Total	1443	40.2	1453	40.5	695	19.3	3591	100	
**G**									
1	67	8.8	107	11.6	32	8.1	206	9.9	**0.041**
2	272	35.6	353	38.4	161	40.9	786	37.8	
3	426	55.7	459	49.9	201	51	1086	52.3	
Total	765	36.8	919	44.2	394	19	2078	100	
**Ki67**									
Median	16		18		18		18		0.811
IQR	9 - 30		10 - 26		10 - 27		10 - 27		
Min - max	0.5 - 95		0.5 - 95		0.5 - 93		0.5 - 95		
Pts with available data	433	36.4	483	40.6	273	23	1189	100	
≤14%	190	43.9	193	40	101	37	484	40.7	0.176
>14%	243	56.1	290	60	172	63	705	59.3	
<20%	236	54.5	268	55.5	143	52.4	647	54.4	0.712
≥20%	197	45.5	215	44.5	130	47.6	542	45.6	
**TILs**									
Median	1		1		5		1		**0.033**
IQR	0 - 5		1 - 5		1 - 5		1 - 5		
Min - max	0 - 80		0 - 80		0 - 60		0 - 80		
Pts with available data	102	37.2	108	39.4	64	23.4	274	100	
**PAM50 subtypes**									
Luminal A	193	28.7	283	49	176	54.2	652	41.4	**<0.001**
Luminal B	127	18.9	162	28	98	30.2	387	24.6	
HER2-enriched	40	5.9	23	4	9	2.8	72	4.6	
Basal-like	294	43.7	88	15.2	32	9.8	414	26.3	
Normal-like	19	2.8	22	3.8	10	3.1	51	3.2	
Total	673	42.7	578	36.7	325	20.6	1576	100	
**IHC subtypes simplified**									
HR-positive	1025	69.6	1296	87.3	641	90.3	2962	80.8	**<0.001**
Triple Negative	448	30.4	189	12.7	69	9.7	706	19.2	

Total	1473	40.3	1485	40.4	710	19.3	3668	100	

Legend and footnotes. Pts: patients; HR: hormone receptors; IQR: interquartile range; IHC: immunohistochemical; TILs: tumor-infiltrating lymphocytes; *: Chi square test for differences in proportions, Kruskalis-Wallis and Wilcoxon rank sum test with continuity correction, where appropriate, for continuous variables (median comparisons)

Supplementary table 2. Demographics according to HR status.

PATIENTS' AND TUMORS' CHARACTERISTICS	HER2-NEGATIVE						P*
	HR-POSITIVE		TRIPLE NEGATIVE		OVERALL		
	N	%	N	%	N	%	
	2962	80.3	706	19.1	3689	100.0	
**Age at diagnosis**							
Median	58.0		51.0		58		**0.001**
IQR	48.0 - 67.0		45.0 - 62.0		48 - 67		
**Sex**							
Male	14	0.5	1	0.1	15	0.4	0.215
Female	2947	99.5	705	99.9	3652	99.6	
Missing	2961	80.7	706	19.3	3667	100.0	
**Menopaual status**							
Pre/perimenopausal	895	35.9	148	47.7	1043	37.2	**<0.001**
Postmenopausal	1596	64.1	162	52.3	1758	62.8	
Missing	2491	88.9	310	11.1	2801	100.0	
**TILs**							
median	1		5		-		0.296
IQR	1 - 5		1 - 40		-		
Pts with available data	269		5		274		
**Histotype**							
Ductal	1563	70.8	279	87.7	1842	73.0	**<0.001**
Lobular	487	22.1	21	6.6	508	20.1	
Other	157	7.1	18	5.7	175	6.9	
Total	2207	87.4	318	12.6	2525	100.0	
**T**							
1	1194	53.0	120	39.0	1314	51.3	**<0.001**
2	776	34.5	133	43.2	909	35.5	
3	184	8.2	27	8.8	211	8.2	
4	98	4.4	28	9.1	126	4.9	
Total	2252	88.0	308	12.0	2560	100.0	
**N**							
0	1332	57.6	157	51.1	1489	56.8	**0.036**
1	646	27.9	87	28.3	733	28.0	
2	183	7.9	34	11.1	217	8.3	
3	152	6.6	29	9.4	181	6.9	
Total	2313	88.3	307	11.7	2620	100.0	
**Metastatic ab initio**							
Yes	330	12.8	36	5.3	366	11.2	**<0.001**
No	2248	87.2	649	94.7	2897	88.8	

Missing	2578	79.0	685	21.0	3263	100.0	
**G**							
1	200	11.0	6	2.4	206	10.0	**<0.001**
2	763	42.0	21	8.3	784	37.9	
3	852	46.9	225	89.3	1077	52.1	
Total	1815	87.8	252	12.2	2067	100.0	
**Ki67**							
≤14%	477	20.7	4	7.4	481	20.4	**0.01**
>14%	676	29.3	23	42.6	699	29.6	
<20%	637	27.6	6	11.1	643	27.2	
≥20%	516	22.4	21	38.9	537	22.8	**0.001**
Missing	2306	97.7	54	2.3	2360	100.0	
**HER2 status**							
IHC 0	1025	34.6	448	63.4	1473	40.2	**<0.001**
IHC 1+	1296	43.8	189	26.8	1485	40.5	
IHC 2+ Not Amplified	641	21.6	69	9.8	710	19.4	
Total	2962	80.8	706	19.2	3668	100.0	
**PAM50 subtyes**							
Luminal A	644	56.6	7	1.6	651	41.4	**<0.001**
Luminal B	385	33.9	1	0.2	386	24.5	
HER2-enriched	35	3.1	37	8.5	72	4.6	
Basal-like	44	3.9	370	84.7	414	26.3	
Normal-like	29	2.5	22	5.0	51	3.2	
Total	1137	72.2	437	27.8	1574	100.0	

Legend and footnotes. *: Chi square test for differences in proportions, Kruskalis-Wallis and Wilcoxon rank sum test with continuity correction, where appropriate, for continuous variables (median comparisons)

Supplementary Table 3. PAM50 intrinsic subtypes distribution within HR-positive and TN tumors according to HER2 status

HR-POSITIVE									
**PAM50 subtypes**	**HER2 0+**		**HER2 1+**		**HER2 2+ NOT AMPLIFIED**		**Overall**		**P***
	**N**	**%**	**N**	**%**	**N**	**%**	**N**	**%**	
Luminal A	187	51.8	283	57.9	174	60.6	644	56.6	**<0.001**
Luminal B	126	34.9	162	33.1	97	33.8	385	33.9	
HER2-enriched	12	3.3	16	3.3	7	2.4	35	3.1	
Basal-like	29	8.0	12	2.5	3	1.0	44	3.9	
Normal-like	7	1.9	16	3.3	6	2.1	29	2.6	
Total	361	31.8	489	43.0	287	25.2	1137	100.0	

TRIPLE NEGATIVE									
**PAM50 subtypes**	**HER2 0+**		**HER2 1+**		**HER2 2+ NOT AMPLIFIED**		**Overall**		**P***
	**N**	**%**	**N**	**%**	**N**	**%**	**N**	**%**	
Luminal A	5	1.6	0	0.0	2	5.4	7	1.6	0.284
Luminal B	1	0.3	0	0.0	0	0.0	1	0.2	
HER2-enriched	28	9.0	7	7.9	2	5.4	37	8.5	
Basal-like	265	85.2	76	85.4	29	78.4	370	84.7	
Normal-like	12	3.9	6	6.7	4	10.8	22	5.0	
Total	311	71.2	89	20.4	37	8.5	437	100.0	

Legend and footnotes. *: Chi square test

Supplementary Table 4. Comparisons of PAM50 intrinsic subtypes distributions within each HER2 IHC score according to HR status

PAM50 subtypes in HER2 0	HR-POSITIVE		TNBC		OVERALL		P*
	HER2 0+		HER2 0+		HER2 0+		
	N	%	N	%	N	%	
Luminal A	187	51.8	5	1.6	192	28.7	<0.001
Luminal B	126	34.9	1	0.3	127	18.9	
HER2-enriched	12	3.3	28	9.0	40	5.9	
Basal-like	29	8.0	265	85.2	294	43.7	
Normal-like	7	1.9	12	3.9	19	2.8	
Total	361	53.7	311	46.3	672	100.0	
PAM50 subtypes in HER2 1+	HER2 1+		HER2 1+		HER2 1+		
	N	%	N	%	N	%	
Luminal A	283	57.9	0	0.0	283	49.0	<0.001
Luminal B	162	33.1	0	0.0	162	28.0	
HER2-enriched	16	3.3	7	7.9	23	4.0	
Basal-like	12	2.5	76	85.4	88	15.2	
Normal-like	16	3.3	6	6.7	22	3.8	
Total	489	84.6	89	15.4	578	100.0	
PAM50 subtypes in HER2 2+ Not Amplified	HER2 2+ Not Amplified		HER2 2+ Not Amplified		HER2 2+ Not Amplified		
	N	%	N	%	N	%	
Luminal A	174	60.6	2	5.4	176	54.2	<0.001
Luminal B	97	33.8	0	0.0	97	30.2	
HER2-enriched	7	2.4	2	5.4	9	2.8	
Basal-like	3	1.0	29	78.4	32	9.8	
Normal-like	6	2.1	4	10.8	10	3.0	
Total	287	88.6	37	11.4	324	100.0	

Legend and footnotes. HR: hormone receptors; **TNBC:** triple negative breast cancer; *: Chi square test

Supplementary table 5. List of genes and subtypes signatures evaluated for differential expression analysis in the overall HER2-negative population and according to HR status

GENE/SIGNATURE	OVERALL	HR-POSITIVE	TNBC
Basal-like	Evaluable	Evaluable	Evaluable
HER2-enriched	Evaluable	Evaluable	Evaluable
Luminal A	Evaluable	Evaluable	Evaluable
Luminal B	Evaluable	Evaluable	Evaluable
Normal-like	Evaluable	Evaluable	Evaluable
ACTR3B	Evaluable	Evaluable	Evaluable
ANLN	Evaluable	Evaluable	Evaluable
AR	Evaluable	Not evaluable	Evaluable
BAG1	Evaluable	Evaluable	Evaluable
BCL2	Evaluable	Evaluable	Evaluable
BIRC5	Evaluable	Evaluable	Evaluable
BLVRA	Evaluable	Evaluable	Evaluable
CCNB1	Evaluable	Evaluable	Evaluable
CCNE1	Evaluable	Evaluable	Evaluable
CD274 (PD-L1)	Evaluable	Not evaluable	Evaluable
CD4	Evaluable	Not evaluable	Evaluable
CD8A	Evaluable	Not evaluable	Evaluable
CDC20	Evaluable	Evaluable	Evaluable
CDC6	Evaluable	Evaluable	Evaluable
CDCA1	Evaluable	Evaluable	Evaluable
CDH3	Evaluable	Evaluable	Evaluable
CENPF	Evaluable	Evaluable	Evaluable
CEP55	Evaluable	Evaluable	Evaluable
CXXC5	Evaluable	Evaluable	Evaluable
EGFR	Evaluable	Evaluable	Evaluable
ERBB2	Evaluable	Evaluable	Evaluable
ESR1	Evaluable	Evaluable	Evaluable
EXO1	Evaluable	Evaluable	Evaluable
FGFR4	Evaluable	Evaluable	Evaluable
FOXA1	Evaluable	Evaluable	Evaluable
FOXC1	Evaluable	Evaluable	Evaluable
GPR160	Evaluable	Evaluable	Evaluable
GRB7	Evaluable	Evaluable	Evaluable
KIF2C	Evaluable	Evaluable	Evaluable
KNTC2	Evaluable	Evaluable	Evaluable
KRT14	Evaluable	Evaluable	Evaluable
KRT17	Evaluable	Evaluable	Evaluable
KRT5	Evaluable	Evaluable	Evaluable
MAPT	Evaluable	Evaluable	Evaluable

MDM2	Evaluable	Evaluable	Evaluable
MELK	Evaluable	Evaluable	Evaluable
MIA	Evaluable	Evaluable	Evaluable
MKI67	Evaluable	Evaluable	Evaluable
MLPH	Evaluable	Evaluable	Evaluable
MMP11	Evaluable	Evaluable	Evaluable
MYBL2	Evaluable	Evaluable	Evaluable
MYC	Evaluable	Evaluable	Evaluable
NAT1	Evaluable	Evaluable	Evaluable
ORC6L	Evaluable	Evaluable	Evaluable
PDCD1 (PD1)	Evaluable	Not evaluable	Evaluable
PGR	Evaluable	Evaluable	Evaluable
PHGDH	Evaluable	Evaluable	Evaluable
PTTG1	Evaluable	Evaluable	Evaluable
RRM2	Evaluable	Evaluable	Evaluable
SFRP1	Evaluable	Evaluable	Evaluable
SLC39A6	Evaluable	Evaluable	Evaluable
TMEM45B	Evaluable	Evaluable	Evaluable
TYMS	Evaluable	Evaluable	Evaluable
UBE2C	Evaluable	Evaluable	Evaluable
UBE2T	Evaluable	Evaluable	Evaluable

Legend and footnotes. HR: hormone receptors; **TNBC:** triple negative breast cancer

Supplementary table 6. All differentially expressed genes of HER2-low vs. HER2 0 tumors in the overall, HR-positive and Triple Negative populations

GENE ID/SIGNATURE	OVERALL		HR-POSITIVE		TNBC	
	Score(d)	FDR*	Score(d)	FDR*	Score(d)	FDR*
Basal-like	-5.67277	**0.0**	-0.00299	24.7	-0.82130	64.3
HER2-enriched	-0.39253	**0.0**	1.18400	**3.2**	-1.30300	64.3
Luminal A	4.73173	**0.0**	3.38402	**0.0**	0.14695	100.0
Luminal B	2.06840	**0.0**	2.34168	**0.0**	-1.22811	64.3
Normal-like	-0.33430	**0.0**	1.14341	**3.2**	-0.07865	68.3
ACTR3B	0.72473	**1.8**	0.74613	8.6	-0.49266	64.3
ANLN	-7.39622	**0.0**	-2.27444	**0.0**	-0.35919	64.3
AR	10.64278	**0.0**	-	-	0.39058	100.0
BAG1	-1.38371	**0.0**	0.90829	6.0	-0.69894	64.3
BCL2	8.20430	**0.0**	2.51291	**0.0**	-0.01781	68.3
BIRC5	-5.55863	**0.0**	-1.40851	**0.9**	-0.27225	68.3
BLVRA	5.14298	**0.0**	1.73118	**0.0**	0.20039	100.0
CCNB1	-3.76904	**0.0**	-1.84185	**0.0**	0.02360	100.0
CCNE1	-9.55346	**0.0**	-3.03781	**0.0**	-0.86694	64.3
CD274 (PD-L1)	2.00646	**0.0**	-	-	-0.42082	64.3
CD4	2.21548	**0.0**	-	-	0.28399	100.0
CD8A	-4.51166	**0.0**	-	-	-0.85870	64.3
CDC20	-4.47577	**0.0**	-0.87565	7.4	-1.06613	64.3
CDC6	-3.37515	**0.0**	-0.28853	21.8	-0.24001	68.3
CDCA1	-4.18599	**0.0**	-1.11544	6.0	-0.81522	64.3
CDH3	-5.87928	**0.0**	-1.06410	6.0	-0.66177	64.3
CENPF	-6.99532	**0.0**	-1.83252	**0.0**	-0.95846	64.3
CEP55	-7.83024	**0.0**	-1.28199	**3.2**	-1.14879	64.3
CXXC5	-3.61475	**0.0**	0.24982	21.8	-0.69201	64.3
EGFR	-0.82066	**0.0**	0.28270	21.8	0.60389	100.0
ERBB2	9.97921	**0.0**	5.17067	**0.0**	1.66991	100.0
ESR1	14.29386	**0.0**	4.96165	**0.0**	1.03532	100.0
EXO1	-7.12534	**0.0**	-2.43602	**0.0**	-1.17480	64.3
FGFR4	-1.39985	**0.0**	-0.07579	24.7	-0.12578	68.3
FOXA1	13.26991	**0.0**	4.92557	**0.0**	0.97629	100.0
FOXC1	-8.43702	**0.0**	-0.73815	9.7	0.20243	100.0
GPR160	7.74052	**0.0**	2.78485	**0.0**	-0.16216	68.3
GRB7	6.09456	**0.0**	2.76279	**0.0**	0.92797	100.0
KIF2C	-3.12355	**0.0**	-1.39427	**0.9**	-0.01905	68.3
KNTC2	-7.77839	**0.0**	-2.31629	**0.0**	-0.72532	64.3
KRT14	-1.86137	**0.0**	0.61877	11.4	0.42407	100.0
KRT17	-5.64524	**0.0**	0.04017	27.5	0.19936	100.0
KRT5	-2.51720	**0.0**	0.84223	7.4	0.79709	100.0
MAPT	9.94512	**0.0**	2.93645	**0.0**	-0.21882	68.3
MDM2	0.06691	5.3	1.12099	**3.2**	-0.21097	68.3
MELK	-6.64057	**0.0**	-1.49359	**0.9**	-0.59390	64.3
MIA	-5.35993	**0.0**	-0.95634	7.4	0.51230	100.0
MKI67	-8.67347	**0.0**	-2.35485	**0.0**	-0.96228	64.3
MLPH	8.78099	**0.0**	2.63999	**0.0**	-0.00518	68.3
MMP11	6.58603	**0.0**	2.60115	**0.0**	0.77140	100.0
MYBL2	-3.97998	**0.0**	-1.44618	**0.9**	-0.90301	64.3
MYC	-5.90597	**0.0**	-0.88871	7.4	-0.01975	68.3
NAT1	12.27258	**0.0**	4.10788	**0.0**	-0.28319	68.3
ORC6L	-7.61981	**0.0**	-2.32482	**0.0**	-0.75740	64.3
PDCD1 (PD1)	0.26237	5.3	-	-	-0.32005	68.3
PGR	11.22998	**0.0**	3.17887	**0.0**	0.52015	100.0
PHGDH	-8.41385	**0.0**	-1.68128	**0.0**	-1.23951	64.3
PTTG1	-4.82226	**0.0**	-1.44424	**0.9**	-0.60645	64.3
RRM2	-5.93472	**0.0**	-1.25960	**3.2**	-0.41814	64.3
SFRP1	-4.83858	**0.0**	-0.33182	16.4	1.23175	100.0

SLC39A6	11.60247	**0.0**	3.96208	**0.0**	-0.38795	64.3
TMEM45B	7.09375	**0.0**	2.02627	**0.0**	0.52291	100.0
TYMS	-3.20623	**0.0**	-0.56947	13.6	-0.49666	64.3
UBE2C	2.37535	**0.0**	-0.37100	16.4	-0.69730	64.3
UBE2T	4.54516	**0.0**	1.69281	**0.0**	-0.55916	64.3

Legend and footnotes. HR: hormone receptors; TNBC: triple negative breast cancer; FDR: false discovery rate; *: significant if FDR<5.0; Score(d): a T-statistic value that reflects a standardized change in expression and measures the strength of the relationship between gene expression and the HER2-low category (versus HER2 0)

Supplementary table 7. Differentially expressed genes among HR-positive/HER2 0, HR-positive/HER2-low, TNBC/HER2 0 and TNBC/HER2-Low

GENE ID	HR+/HER2 0 *Contrast*	HR+/HER2-low *Contrast*	TNBC/HER2 0 *Contrast*	TNBC/HER2-low *Contrast*	FDR*
ESR1	6.861742068	11.1074889	-12.90998321	-12.08492615	**0**
FOXA1	5.778217896	8.825351095	-10.4809953	-9.413459819	**0**
NAT1	4.318943613	8.027254553	-8.897426094	-9.080491687	**0**
SLC39A6	3.964146625	7.87369166	-8.600693487	-8.83063485	**0**
PGR	3.688389562	6.507937351	-7.385485991	-7.0821826	**0**
AR	3.652669581	6.241609361	-7.154953824	-6.753890554	**0**
MAPT	3.542191338	5.97143271	-6.749174571	-6.935422423	**0**
FOXC1	-4.981864542	-5.538845927	6.9775032	7.185258765	**0**
CCNE1	-3.37762021	-6.018579053	6.943340875	6.038719686	**0**
MLPH	2.581366165	4.571432528	-5.139368572	-5.143849516	**0**
PHGDH	-3.455515264	-4.906927001	6.05145693	4.868929478	**0**
MKI67	-2.985374693	-5.147177429	5.996903821	5.137457962	**0**
BCL2	3.004496715	5.226004989	-5.895454984	-5.913599185	**0**
CEP55	-3.612167568	-4.800907334	6.001214003	4.933726234	**0**
GPR160	2.295959541	4.559067178	-4.97306946	-5.141532706	**0**
ERBB2	0.853469611	5.811851031	-5.865658229	-4.065682234	**0**
KRT17	-3.053703742	-3.024321445	3.939545973	4.115344828	**0**
KNTC2	-2.521227666	-4.615649371	5.312815869	4.533560183	**0**
ANLN	-2.394303094	-4.430910963	5.010002631	4.649030645	**0**
ORC6L	-2.315621728	-4.553831424	5.153881435	4.455539376	**0**
TMEM45B	2.195984431	3.7004643	-4.316099686	-3.778937655	**0**
CENPF	-2.356851503	-3.977967627	4.718493603	3.747639361	**0**
SFRP1	-2.673552292	-2.925551032	3.491589308	4.638618441	**0**
MELK	-2.567267617	-3.980373357	4.725372162	4.171204745	**0**
MYC	-2.623452681	-3.435772806	4.15842533	4.142345807	**0**
MIA	-2.348514667	-2.976385496	3.519190449	4.071299962	**0**
EXO1	-1.689980966	-3.92999769	4.430571237	3.267594746	**0**
CDH3	-2.183753512	-3.074722651	3.770177997	3.174202065	**0**
RRM2	-2.207292008	-3.398663576	4.016121429	3.666137082	**0**
MMP11	1.018291318	3.028611945	-3.254201613	-2.548210338	**0**

BIRC5	-1.847675311	-3.098541079	3.577831485	3.323902063	**0**
GRB7	0.736362322	3.304204501	-3.433618431	-2.451544203	**0**
CXXC5	-1.912486573	-1.700216734	2.397390797	2.01123729	**0**
BLVRA	1.572120525	3.282508268	-3.616414509	-3.393016952	**0**
KRT5	-2.145753017	-1.520262446	2.119765859	2.802387954	**0**
UBE2T	1.104568388	2.625459449	-2.684460847	-3.204873418	**0**
PTTG1	-1.357405686	-2.746825946	3.129379599	2.524954233	**0**
CDC20	-1.561003057	-2.341316049	2.956453401	1.870697613	**0**
CD8A	-1.06424082	-2.222143957	2.587451424	1.75430237	**0**
CDCA1	-1.221013694	-2.220418771	2.658470223	1.797942709	**0**
CDC6	-1.826050077	-2.119404114	2.698602991	2.469801753	**0**
UBE2C	1.935072987	1.613637076	-2.113706106	-2.78929955	**0**
KRT14	-1.484755864	-1.07590958	1.498947303	1.917394719	**0**
MYBL2	-0.566292389	-1.883199658	2.063921511	1.287040354	**0**
TYMS	-1.245558217	-1.757831987	2.189600191	1.675491781	**0**
CCNB1	-0.414304024	-2.224612346	2.136840048	2.161647332	**0**
KIF2C	-0.435803157	-1.716742224	1.706395887	1.687281857	**0**
BAG1	-1.591018957	-0.745488035	1.416825439	1.030570598	**0**
CD4	1.278682017	1.372164069	-1.860963503	-1.357264788	**0**
CD274 (PD-L1)	0.750358454	1.166321248	-1.32500004	-1.450554218	**0**
EGFR	-0.885894045	-0.653610577	0.831535115	1.439230248	**0**
FGFR4	-0.62915164	-0.693176137	0.901706886	0.800116908	**0**
MDM2	-1.186448168	-0.079815829	0.607015399	0.460937749	**0.1**
ACTR3B	-0.247048402	0.516653373	-0.24331634	-0.769816605	**1.8**
PDCD1 (PD1)	0.229962949	0.162491103	-0.235653338	-0.265422996	**3.0**

Legend and footnotes. FDR: false discovery rate; *: significant if FDR<5.0; HR: hormone receptor; TNBC: triple negative breast cancer; *Contrast*: is the standardized mean difference between the gene's expression in a class vs. its overall mean expression in the overall dataset.

Supplementary Table 8. Gene expression of HER2-2+ compared to HER2 1+ and 0 tumors in the HR-positive subset

GENE/SIGNATURE	HR-POSITIVE/HER2-NEGATIVE			
	HER2 0 *Contrast*	HER2 1+ *Contrast*	HER2 2+ NA *Contrast*	FDR*
Basal-like	0.00283	-0.88603	1.45201	**3.6**
HER2-enriched	-1.16229	-0.44507	2.21633	**0.0**
Luminal A	-3.21810	0.46473	3.34852	**0.0**
Luminal B	-2.25388	-0.16542	3.15172	**0.0**
Normal-like	-1.05603	-0.39148	1.99252	**0.9**
ACTR3B	-0.58029	0.17146	0.45979	14.1
ANLN	1.57948	-0.47296	-1.24122	**0.0**
BAG1	-0.53348	-0.54786	1.58172	**0.0**
BCL2	-1.75095	0.37398	1.62293	**0.0**
BIRC5	0.93034	-0.64932	-0.12203	**3.6**
BLVRA	-1.36454	0.36271	1.14771	**0.9**
CCNB1	1.38774	-0.34588	-1.20499	**0.0**
CCNE1	2.22623	-0.36631	-2.24283	**0.0**
CDC20	0.61235	-0.68690	0.34603	7.2
CDC6	0.20957	-0.39872	0.38725	17.9
CDCA1	0.79716	-0.32239	-0.48896	7.6
CDH3	0.66493	-0.06819	-0.73761	7.2
CENPF	1.26956	-0.66734	-0.52587	**0.9**
CEP55	0.87016	-0.94497	0.44058	**2.3**
CXXC5	-0.13594	-0.42676	0.87480	**3.6**
EGFR	-0.18705	0.13757	0.01300	23.1
ERBB2	-3.94793	0.94579	3.49078	**0.0**
ESR1	-2.98901	0.75918	2.57206	**0.0**
EXO1	1.70203	-0.46725	-1.40718	**0.0**
FGFR4	0.04529	-0.26643	0.37983	17.9
FOXA1	-3.05763	0.93147	2.37671	**0.0**
FOXC1	0.47400	-0.61673	0.40753	7.2
GPR160	-1.88797	0.16030	2.14905	**0.0**
GRB7	-2.03291	0.49749	1.78031	**0.0**
KIF2C	0.98070	-0.63800	-0.20498	**3.6**
KNTC2	1.61274	-0.99690	-0.42297	**0.0**
KRT14	-0.35626	0.27656	0.00087	17.9
KRT17	-0.02275	0.23574	-0.35823	17.9
KRT5	-0.47377	0.25375	0.18850	14.1
MAPT	-1.77205	0.44702	1.52990	**0.0**
MDM2	-0.71511	0.04468	0.84036	**3.6**
MELK	1.02701	-0.83862	0.06545	**2.3**
MIA	0.59910	-0.58990	0.20360	7.2
MKI67	1.55384	-0.95257	-0.42053	**0.0**
MLPH	-1.51625	-0.02501	1.97851	**0.0**
MMP11	-1.55869	0.86881	0.56434	**0.0**
MYBL2	0.93321	-0.29141	-0.71369	**3.6**
MYC	0.56299	-0.63562	0.32485	7.2
NAT1	-2.40914	0.90353	1.59398	**0.0**
ORC6L	1.60517	-0.95927	-0.47510	**0.0**
PGR	-1.79970	0.93024	0.77136	**0.0**
PHGDH	1.10544	-0.67271	-0.30734	**2.3**
PTTG1	1.04078	-0.94181	0.21738	**2.3**
RRM2	0.82596	-0.84250	0.32871	**2.3**
SFRP1	0.19926	0.37772	-0.87516	7.2
SLC39A6	-2.47832	0.13383	2.94689	**0.0**
TMEM45B	-1.28927	0.80674	0.32204	**0.0**
TYMS	0.40551	-0.74150	0.70003	7.2
UBE2C	0.24692	-0.39737	0.33732	14.1
UBE2T	-1.13151	-0.22770	1.81989	**0.0**

Legend and footnotes. Neg: negative; **ISH:** in-situ hybridization; **HR:** hormone receptors; **NA:** not amplified; **FDR:** false discovery rate; *: significant if FDR<5.0; *Contrast*: is the standardized mean difference between the gene's expression in a class vs. its overall mean expression in the overall dataset.

Supplementary table 9. Included studies' description

Study	Study Centers	Setting	Study type	N. of pts	Tumor types included	PAM50 type	Study reference
Cooperational spanish-italian study (including patients from the GEICAM/2012-09 study)	15 hospitals across Spain (centralized Prosigna assay and centralized pathology re-evaluation at the Gregorio Marañón Biomedical Research Institute, Madrid)	Early (Adjuvant)	Observational prospective	194	HR+/HER2-	Commercialized and standardized PAM50/Prosigna assay (NanoString Technologies, Seattle, WA) on FFPE tissues	Martin M, et al. Curr Med Res Opin. 2015; 31:1129-1137 and Fernandez-Martinez, A. et al. Oncotarget 8, 21930–21937 (2017)
	Campus Bio-Medico University (Rome)	Early (Adjuvant)	Observational prospective	159	HR+/HER2-		Fernandez-Martinez, A. et al. Oncotarget 8, 21930–21937 (2017) + unpublished
	Vall d'Hebron Institute of Oncology (Barcelona)	Early (Adjuvant)	Observational prospective	117	HR+/HER2-		Fernandez-Martinez, A. et al. Oncotarget 8, 21930–21937 (2017).
c-Bioportal	A patient-driven initiative on the Metastatic Breast Cancer Project platform	Early & Metastatic	Observational prospective	103	HR+/HER2- and TNBC	N/A	The Metastatic Breast Cancer Project. https://www.mbcproject.org/
	Memorial Sloan Kettering Cancer Center	Early	Observational retospective	1637	HR+/HER2- and TNBC	N/A	Razavi, P. et al. Cancer Cell 34, 427-438.e6 (2018).
	TCGA	Early	Observational retospective	111	HR+/HER2- and TNBC	Intrinsic subtypes were defined by applying the PAM50 predictor fom Parker, J. S. et al. J. Clin. Oncol. 27, 1160–1167 (2009)	Ciriello, G. et al. Cell 163, 506–519 (2015).
	TCGA	Early	Observational retospective	207	HR+/HER2- and TNBC	N/A	Cancer Genome Atlas Network. Nature 490, 61–70 (2012).
SOLTI 1501-VENTANA	9 hospitals in Spain (including the Hospital Clinic of Barcelona and the Vall d'Hebron Institute of Oncology)	Early (Neoadjuvant)	Randomized window-of-opportunity	46	HR+/HER2-	Breast 360TM Codeset for PAM50 assay (NanoString Technologies, Seattle, WA) on FFPE tissues. Intrinsic molecular subtypes were identified using the research-based PAM50 predictor described in Prat A, et al. JAMA Oncol 2016; 2: 1287–94 and Vidal M, et al. Mol Oncol 2015; 9: 1081–90.	Adamo, B. et al. Breast Cancer Res. 21, 108 (2019).

SOLTI 1402-CORALEEN	21 hospitals in Spain (only the Hospital Clinic of Barcelona cohort was used)	Early (Neoadjuvant)	Randomized phase II	14	HR+/HER2-	Commercialized and standardized PAM50/Prosigna assay (NanoString Technologies, Seattle, WA) on FFPE tissues	Prat, A. et al. Lancet Oncol. 21, 33–43 (2020).
CIBOMA/2004-01_GEICAM/2003-11 study	80 institutions from Spain, Brazil, Chile, Colombia, Ecuador, Mexico, Peru, and Venezuela (pathology centralized)	Early (Adjuvant)	Randomized phase III	375	TNBC		Lluch, A. et al. J. Clin. Oncol. 38, 203–213 (2020).
Hospital Clinic of Barcelona internal databases	Hospital Clinic of Barcelona	Early & Metastatic	Observational prospective and retrospective	726	HR+/HER2- and TNBC	Commercialized and standardized PAM50/Prosigna assay (NanoString Technologies, Seattle, WA) on FFPE tissues and research-based PAM50 predictor described in Prat A, et al. JAMA Oncol 2016; 2: 1287–94 and Vidal M, et al. Mol Oncol 2015; 9: 1081–90.	N/A

Legend and footnotes. N/A: not applicable; **HR+:** hormone receptor positive: **-:** negative; **TNBC:** triple negative breast cancer; **FFPE:** formalin-fixed paraffin-embedded; pts: patients.

Supplementary figures

Supplementary figure 1. Plot for 2 class unpaired SAM analysis in the overall population

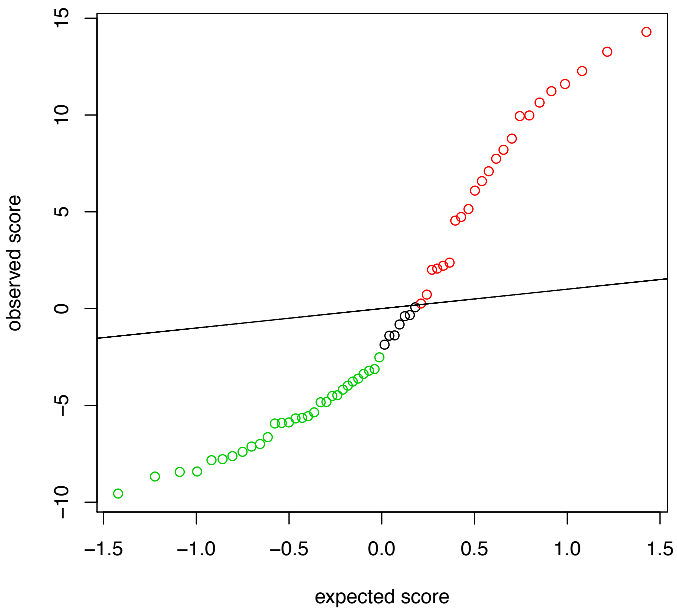

Legend: The red dots represent relative high gene expression, the green dots represent relative low gene expression, and black dots represent median gene expression for HER2-low vs. HER2 0 in the overall population.

Supplementary figure 2. Plot for 2 class unpaired SAM analysis in the HR-positive population

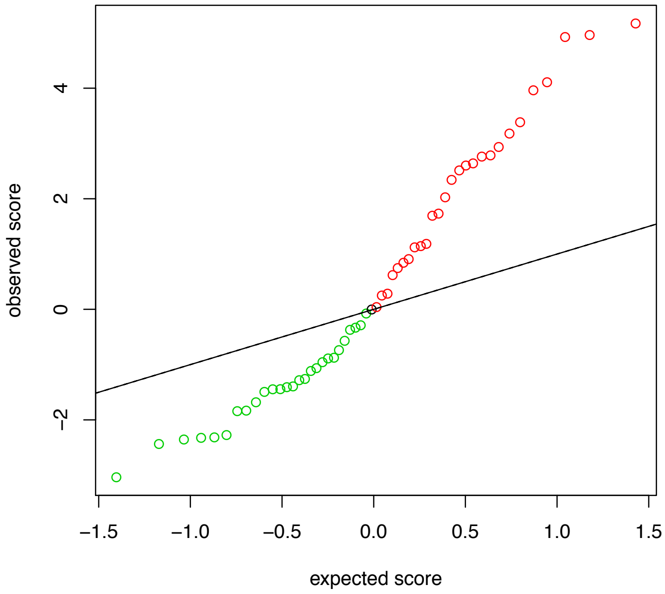

Legend: The red dots represent relative high gene expression, the green dots represent relative low gene expression, and black dots represent median gene expression for HER2-low vs. HER2 0 in the HR-positive population.

Supplementary figure 3. Plot for 2 class unpaired SAM analysis in the TN population

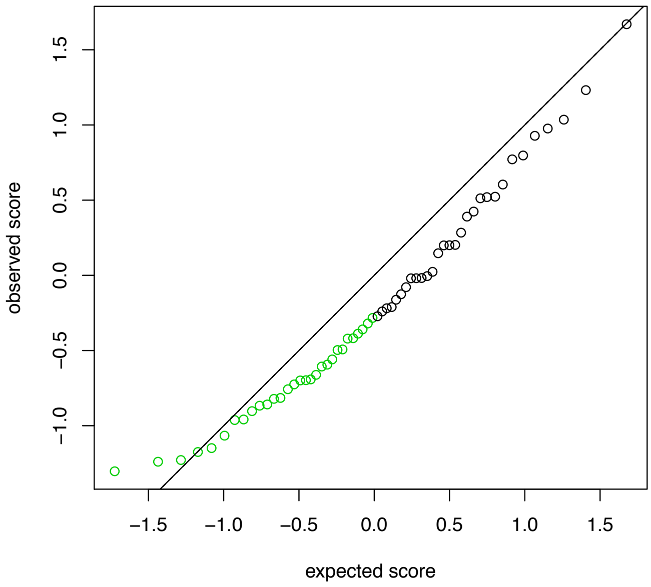

Legend: The green dots represent relative low gene expression, and black dots represent median gene expression for HER2-low vs. HER2 0 in the TNBC population.

Supplementary figure 4. Gene expression patterns of the HR-positive/HER2-negative breast cancers

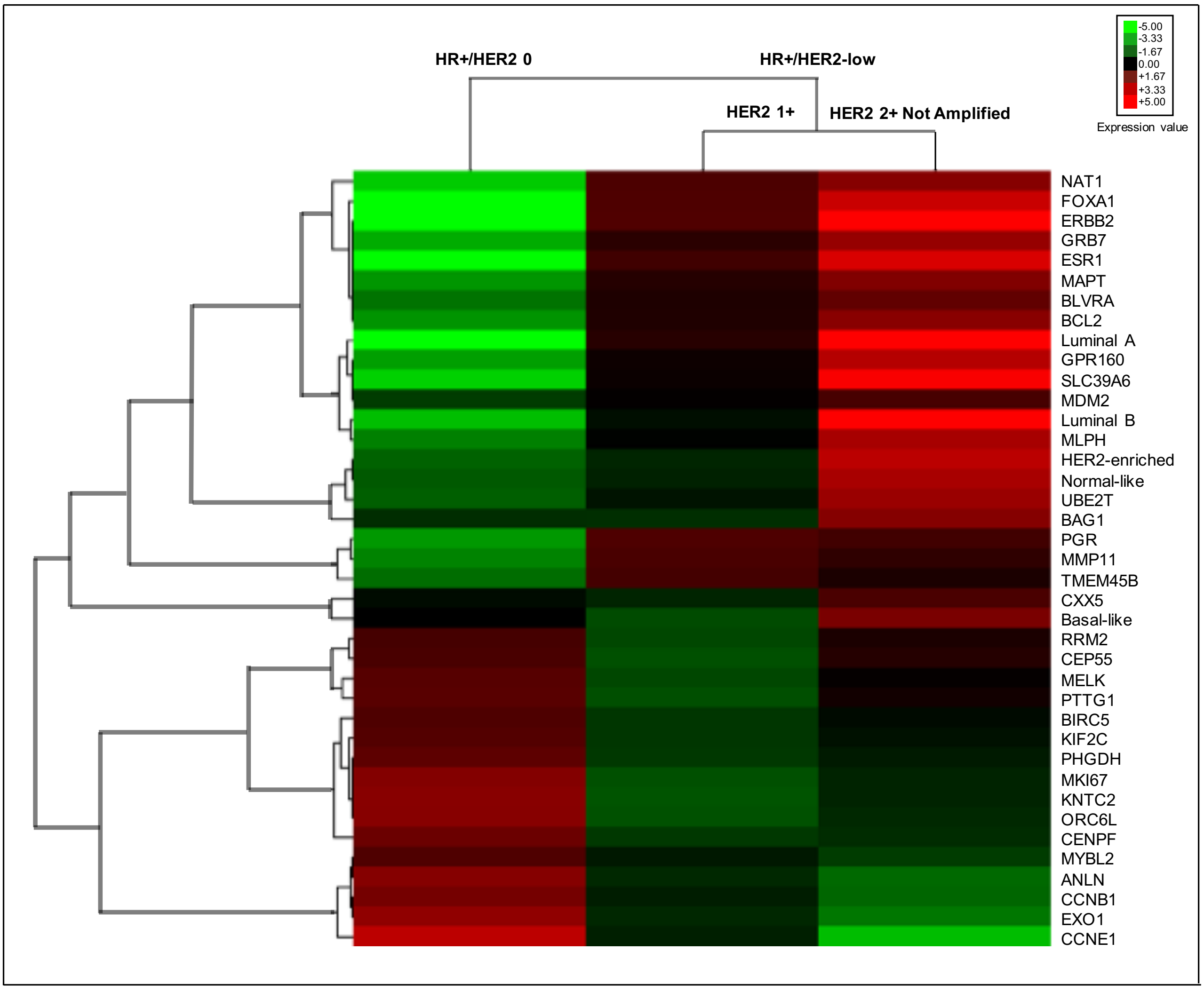

Legend. A: supervised cluster of the HR-positive/HER2-negative breast cancers (total of 3 classes according to HER2 IHC score) with 55 variables. Sample and gene expression data from tumor samples of the same subtype have been combined into a single category. For each gene in a class, we calculated the standardized mean difference between the gene's expression in that class vs. its overall mean expression in the dataset using a 3-class Significance Analyses of Microarrays. The red color represents relative high gene expression, green represents relative low gene expression, and black represents median gene expression. **HR:** hormone receptors.

Supplementary Data Set

Anonymized database with already normalized patients' PAM50 gene expression data and their hormone receptor status, HER2 immunohistochemical score, HER2-low status and intrinsic subtype.
